# Cambium LBDs promote radial growth by regulating PLL-mediated pectin metabolism

**DOI:** 10.1038/s41477-025-02151-1

**Published:** 2025-11-14

**Authors:** Lingling Ye, Xin Wang, Juan José Valle-Delgado, Julia P. Vainonen, Isaac Wopereis, Kavindra Kumar Kesari, Junko Takahashi, Maija Sierla, Ari Pekka Mähönen

**Affiliations:** 1 Organismal and Evolutionary Biology Research Programme, Faculty of Biological and Environmental Sciences and Viikki Plant Science Centre, University of Helsinki, Helsinki, Finland; 2 Department of Bioproducts and Biosystems, School of Chemical Engineering, Aalto University, Espoo, Finland; 3 Cluster of Plant Developmental Biology, Laboratory of Cell and Developmental Biology, Wageningen University & Research, Wageningen, the Netherlands; 4 Department of Forest Genetics and Plant Physiology, Umeå Plant Science Centre, Swedish University of Agricultural Sciences, Umeå, Sweden; 5 Present Address: Institute of Emerging Agricultural Technology, Shenzhen University of Advanced Technology, Shenzhen, China; 6 Present Address: Faculty of Synthetic Biology, Shenzhen University of Advanced Technology, Shenzhen, China; 7 Present Address: Renewable and Sustainable Energy Research Center, Technology Innovation Institute, Abu Dhabi, United Arab Emirates

**Keywords:** Plant morphogenesis, Cell wall, Transgenic plants, Cytokinin

## Abstract

Plant growth originates from the interlinked action of cell division and cell growth. During radial growth of secondary tissues, bifacial cambial stem cells grow and divide to produce xylem and phloem precursors, which subsequently undergo expansion characteristic of their respective differentiation processes. In *Arabidopsis* roots, cytokinins and four downstream LATERAL ORGAN BOUNDARIES DOMAIN (LBD) transcription factors are key players in promoting radial growth, though the underlying mechanisms remain unknown. Here our results indicate that these LBD genes primarily regulate cell growth rather than proliferation. Through a large-scale CRISPR–Cas9-aided reverse genetic screen, we identified a set of *PECTATE LYASE-LIKE* (*PLL*) genes that function downstream of cytokinin and the LBDs in the regulation of radial growth. We show that at least one of these PLLs, PLL18, possesses pectate lyase activity. In accordance with this activity, PLLs and LBDs promote radial growth by modifying the pectin composition and mechanical properties of the primary cell wall. Our findings thus connect the central role of cytokinins in radial growth with cell wall remodelling and pave a way for further research on hormone-mediated plant growth regulation and cell wall metabolism.

## Abstract (web summary)

This study reveals that LBD transcription factors in the cambium drive radial plant growth by regulating PECTATE LYASE-LIKE (PLL) enzymes that remodel cell wall pectin, promoting cell expansion.

## Main

Plant growth originates from cell growth (that is, increase in cell size) and divisions. Unlike animal cells, plant cells are surrounded by strong yet extensible primary walls rich in polysaccharides such as cellulose, hemicellulose and pectin. To coordinate cell division and cell growth, a delicate regulation of cell wall metabolism is required. Cell-wall-loosening agents are thought to regulate cell growth by promoting wall stress relaxation, thus leading to turgor reduction that triggers water uptake and cell enlargement, ultimately restoring turgor and wall stress^1^. The best-characterized agents are expansins, which are proposed to promote cell enlargement non-enzymatically by disrupting the non-covalent binding of wall cellulose^2^. Early studies on expansins have largely relied on overexpression analysis and characterized their activities in vitro^2,3^, and their biological importance in various developmental contexts is only beginning to emerge^2^. Another type of potential cell-wall-loosening agents are the xyloglucan endotransglucosylase/hydrolases (XTHs). XTHs cut and ligate xyloglucan, the primary form of hemicellulose that interconnects cellulose microfibrils^1,4^. Cellulose and hemicellulose are embedded in a gel-like matrix of pectins, which play important roles in cell wall structure by affecting cell wall assembly, mechanics and growth. Moreover, cell wall pectins serve not only as structural components but also as signalling molecules in wall integrity signalling^1,5–7^. Pectin-metabolism-related agents, such as polygalacturonases, pectate lyases, pectin methylesterases (PMEs) and PME inhibitors (PMEIs), are therefore widely implicated in the regulation of plant growth and development^5,8–11^, including vascular development^12–14^, thus representing another type of cell-wall-loosening agent. However, since these potential cell-wall-loosening agents are typically present in a highly redundant manner in plant genomes, their roles in controlling cell growth have been identified in only a few family members. A systematic characterization of their functions in cell growth regulation and consequently in development is therefore needed.

In the secondary tissues of *Arabidopsis* roots, radial growth mediated by bifacial cambial stem cells involves two main processes: first, meristematic cell growth in the radial dimension, coupled with periclinal cell divisions, which produce daughter cells with xylem and phloem identity in opposite directions; and second, radial cell expansion associated with differentiation, providing further radial growth, particularly for phloem parenchyma cells and xylem vessels^15,16^. Our previous study identified four plant-specific LATERAL ORGAN BOUNDARIES DOMAIN (LBD) transcription factors, LBD1, LBD3, LBD4 and LBD11 (here termed CAMBIUM LBDs (CALBDs)), that regulate both vascular cell proliferation and cell growth downstream of cytokinin signalling, the central plant hormone promoting cambial activity^17,18^. Here we proceeded to explore the molecular targets of CALBDs in regulating radial growth of the *Arabidopsis* root. Through transcriptomic analysis followed by a large-scale CRISPR–Cas9-aided reverse genetic screen, we identified a set of genes encoding PECTATE LYASE-LIKE (PLL) proteins that function downstream of cytokinins and CALBDs to regulate radial growth at both the cellular and organ levels. Our results suggest that CALBDs and PLLs mediate radial growth, probably by modulating the pectin composition and mechanical properties of primary cell walls. These findings highlight the dynamic and complex nature of primary cell walls and their influence on radial growth in secondary tissues.

## Results

### CALBDs predominantly regulate cell growth rather than cell divisions

**Fig. 1:**
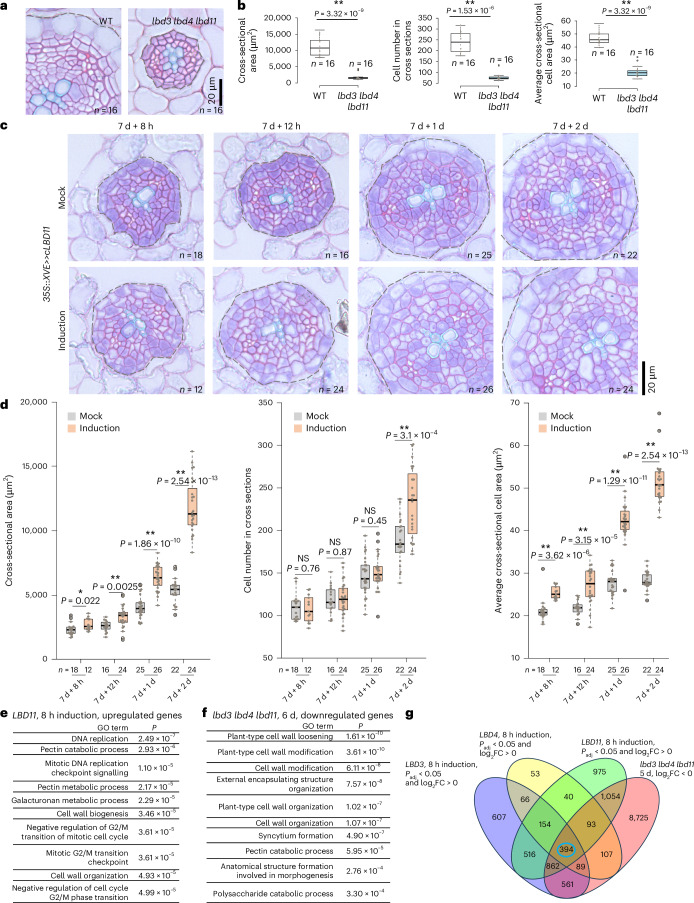
*CALBDs* promote cross-sectional cell growth during root secondary growth. **a**, Plastic cross sections of nine-day-old WT and *lbd3* *lbd4* *lbd11* roots, taken 5 mm below the hypocotyl–root junction. **b**, Quantification of cross-sectional area, cell number in cross sections and average cross-sectional cell area for WT and *lbd3* *lbd4* *lbd11* roots from the experiment in **a**. **c**, Plastic cross sections of *LBD11* inducible overexpression lines. Seven-day-old roots of the *LBD11* inducible overexpression line (*35S::XVE*>>*cLBD11*) were treated for 8 hours, 12 hours, 1 day or 2 days (left to right) with either mock treatment or 17-β-oestradiol treatment. **d**, Quantification of cross-sectional area, cell number in cross sections and average cross-sectional cell area for WT and *lbd3* *lbd4* *lbd11* roots from the experiment in **c**. The cells and area inside the dashed lines (**a**,**c**) were considered in the quantifications of cell number in cross sections and cross-sectional area, respectively (Extended Data Fig. 1a). Statistical significance was assessed using a two-tailed Mann–Whitney test (**b**,**d**). *P < 0.05; **P < 0.01; NS, not significant. *n* and the grey dots within the box plots represent individual roots used in each analysis (**a**–**d**). The data in **b**,**d** are presented as box plots showing the median (centre line), the interquartile range (IQR) (box boundaries; 25th and 75th percentiles) and whiskers extending to the minimum and maximum values within 1.5 × IQR of the quartiles. **e**, GO enrichment analysis of the most differentially upregulated genes (*P*_adj_ < 0.05; log_2_FC > 1) in *LBD11* inducible overexpression lines after eight hours of induction. FC, fold change. **f**, GO enrichment analysis of significantly downregulated genes (*P*_adj_ < 0.05) in the *lbd3* *lbd4* *lbd11* mutant at six days old. One-sided hypergeometric tests were used (**e**,**f**). **g**, Venn diagram showing the overlapping significantly differentiated genes that are upregulated in all *LBD3*, *LBD4* and *LBD11* overexpression lines with eight hours of induction, and downregulated in five-day-old *lbd3* *lbd4* *lbd11* mutants. Source data

**Extended Data Fig. 1:**
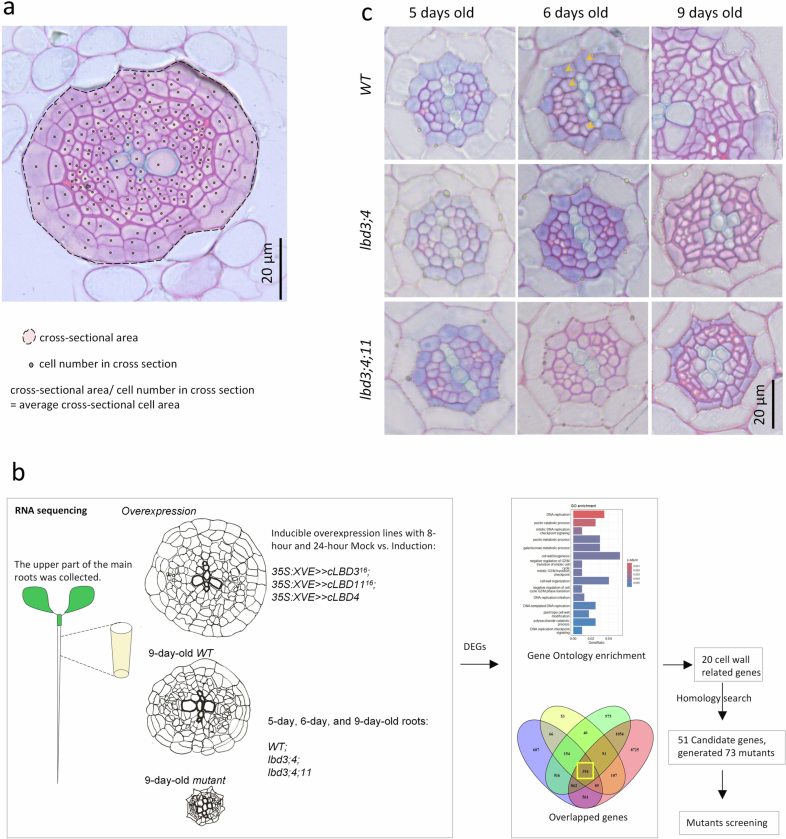
Illustration of cross-sectional area and cross-sectional cell growth in *Arabidopsis* roots along with a workflow for candidate gene identification. **a**, The diagram illustrates the measurement of cross-sectional area, cell number in cross section, and cross-sectional cell growth used in this study. Cells and areas within the dashed line were included in the quantifications of cell number in cross section and cross-sectional area, respectively. Average cross-sectional cell area was calculated by dividing cross-sectional area by cell number in cross section. **b**, Schematic workflow for identifying cell wall modification genes during root radial growth. **c**, Plastic cross-sections of *WT*, *lbd3;4*, and *lbd3;4;11* roots at 5, 6, and 9 days old. Arrowheads indicate cell divisions in the procambium and pericycle, marking the onset of secondary growth in *WT*, which is delayed in *lbd* mutants.

In our previous study, we identified CALBDs as essential regulators of radial growth downstream of cytokinin signalling in the secondary tissues of *Arabidopsis* roots^17^. While CALBDs are required for both periclinal cell divisions and radial cell growth (hereafter referred to as cell growth), analyses of knockout mutants and inducible overexpression lines suggested that CALBDs primarily regulate cell growth^17^ (Fig. 1a–d). To investigate the roles of CALBDs in cell division and cell growth in detail, we performed a time-course induction experiment for the *LBD11* inducible overexpression line. We compared cell number and average cell area in cross sections of secondary tissues between induced samples and mock-treated controls (Fig. 1c–d and Extended Data Fig. 1a). An eight-hour induction was sufficient to significantly trigger cell growth in the secondary tissues of seven-day-old *Arabidopsis* roots, while the cell number remained the same as in the mock treatment. Extending the induction time to 12 hours or one day resulted in further cell growth, but the cell number still did not differ from the mock treatment. A significant increase in cell number, compared with the mock treatment, was observed only after a two-day induction (Fig. 1c,d). These findings suggest that CALBDs predominantly promote cell growth in the secondary tissue, which is essential to compensate for the halving of cell volume that occurs after each cambial stem cell division. The reduced number of cells in cross sections observed in *calbd* mutants is probably due to the prolonged time required for cambial cells to reach the threshold volume necessary for division, similar to the cell size control in the shoot apical meristem^19^.

**Extended Data Fig. 2:**
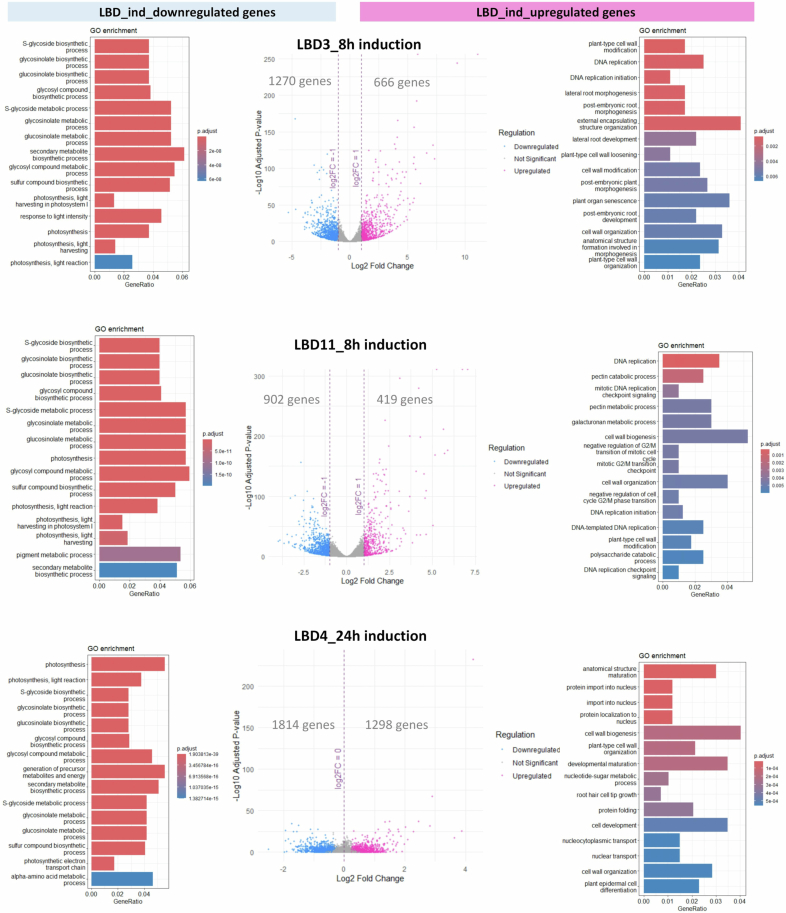
GO enrichment analysis in *LBD3*, *LBD4*, *LBD11* inducible overexpression lines. For *LBD3* and *LBD11* inducible overexpression, genes used for GO enrichment were identified using a threshold of padj < 0.05 and a fold change greater than 2 (|log2FC | > 1), based on 8 hours of induction. One-sided hypergeometric test was applied. For *LBD4* inducible overexpression, only padj < 0.05 was applied for GO analysis, with data from 24 hours of induction presented, as *LBD4* overexpression shows phenotypic changes primarily after extended induction^17^.

**Extended Data Fig. 3:**
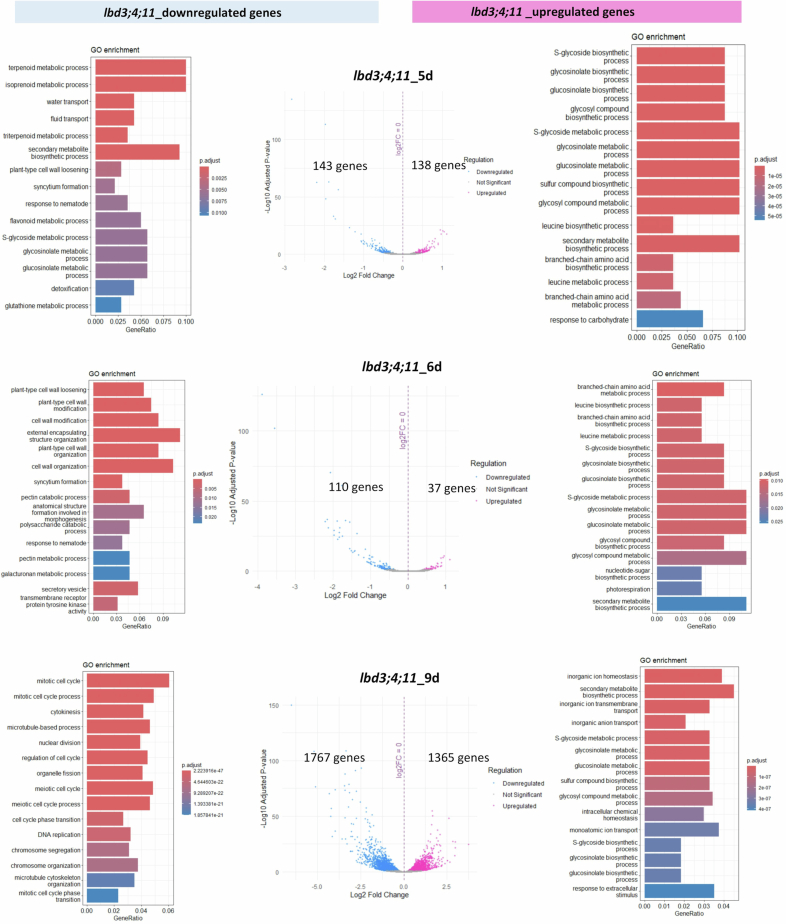
GO enrichment analysis in the *lbd3;4;11* mutant at 5, 6, and 9 days old, respectively. Significantly expressed genes used for GO enrichment analysis were identified with a threshold of padj < 0.05. One-sided hypergeometric test was applied.

To elucidate the molecular mechanism underlying CALBD-mediated cell growth, we performed transcript profiling on root samples of both *CALBD* inducible overexpression lines and *calbd* mutants (Extended Data Fig. 1b). This analysis included previously published transcriptomic data from inducible overexpression lines of *LBD3* and *LBD11* (ref. ^17^) and newly generated data from an *LBD4* inducible overexpression line following 8-hour and 24-hour inductions. Additionally, we analysed wild-type (WT) plants and *lbd3* *lbd4* and *lbd3* *lbd4* *lbd11* mutants at five, six and nine days old, spanning stages before and after the onset of the root radial growth phenotype (Extended Data Fig. 1b,c). Analysis of the combined dataset revealed that Gene Ontology (GO) terms associated with primary cell wall modification were significantly overrepresented among the most upregulated genes following an 8-hour induction of *LBD3* and *LBD11* (Fig. 1e and Extended Data Fig. 2) and after a 24-hour induction of *LBD4* (Extended Data Fig. 2). Conversely, these cell-wall-related GO terms were underrepresented in the *calbd* mutants, even in five- and six-day-old roots (Fig. 1f and Extended Data Fig. 3), prior to the appearance of an obvious radial growth phenotype. While GO terms related to cell cycle regulation and DNA replication were upregulated as early as eight hours post-induction (Fig. 1e and Extended Data Fig. 2), accelerated cell divisions were observed only after two days of induction (Fig. 1c,d). Moreover, cell-division-related GO terms were not underrepresented in five- and six-day-old *calbd* mutant roots. Thus, while we cannot entirely rule out the possibility that CALBDs also regulate cell division, our data strongly suggest that CALBDs primarily promote radial cell growth, probably by modulating primary cell wall composition.

### A CRISPR–Cas9-aided reverse genetic screen identified a set of PLLs as positive regulators of radial growth

**Extended Data Fig. 4:**
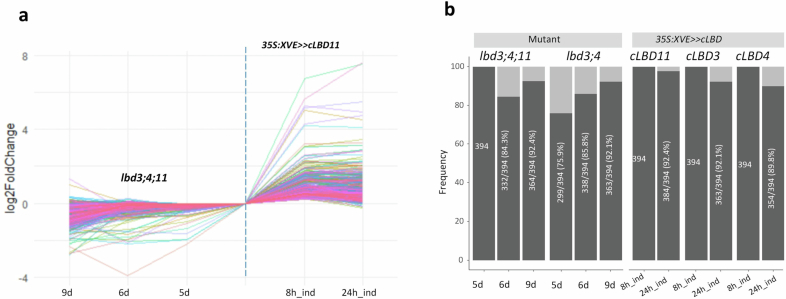
Expression analysis of overlapping genes at different stages. **a**. Line plot showing the log2FoldChange of 394 overlapping genes in the LBD11 inducible overexpression line following 8-hour and 24-hour induction, as well as their relative expression levels in 5-day-old, 6-day-old, and 9-day-old *lbd3;4;11* mutants. **b**. The ratio of the genes maintaining the downregulated trend as observed in 5-day-old *lbd3;4;11* mutants (left); and the ratio of the genes maintaining the upregulated trend after 8 hours of induction in the overexpression line (right).

To identify the downstream targets of CALBDs, we first examined genes that were significantly upregulated by LBD3, LBD4 and LBD11 after eight hours of induction and downregulated in the five-day-old *lbd3* *lbd4* *lbd11* mutant, which shows radial growth comparable to that of the WT root (Fig. 1g). This analysis yielded 394 candidate genes (Fig. 1g and Supplementary Table 1). Over 80% of these genes remained consistently differentially expressed in overexpression lines after 24 hours of induction and in mutants at later stages (Extended Data Fig. 4), suggesting their role in CALBD-mediated regulation of radial growth across developmental stages. Given the impact of CALBDs on cell growth, we refined our focus to candidates associated with primary cell wall metabolism. We prioritized candidate genes encoding potential cell-wall-loosening agents^1,4^, such as genes encoding expansins, XTHs, GLYCOSIDE HYDROLASEs and pectin metabolism enzymes such as PMEs, PMEIs and PLLs. To cope with the likely redundancy, we also considered their homologous genes outside of these 394 candidates that showed transcription in secondary tissues (RNA-seq reads greater than ten in any WT sample at five, six or nine days) and significant transcriptional changes in at least one profiling dataset. Several cell-wall-related genes that lack clear functional annotations but were highly represented in the profiling data were also included. A total of 51 genes were selected for subsequent reverse genetic screening (Supplementary Table 2). We performed a CRISPR–Cas9-aided gene editing screen^20^, resulting in 73 mutation combinations across 51 candidate genes (Extended Data Fig. 1b and Supplementary Table 3). These knockout mutants were then phenotyped for radial growth.

**Fig. 2:**
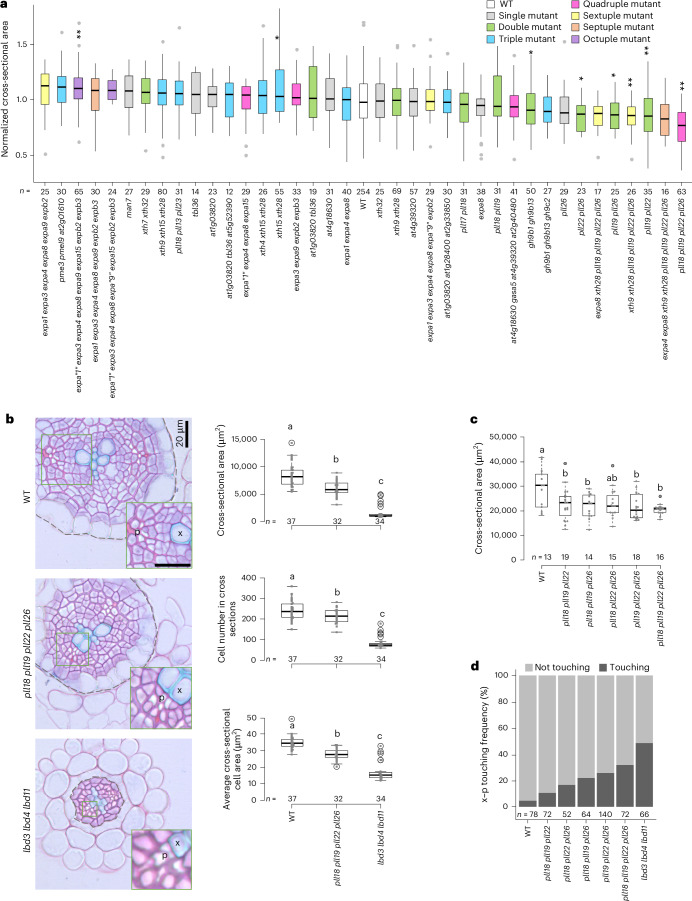
A set of *PLL* genes are required for root secondary growth, identified through CRISPR–Cas9 mutant screening. **a**, Box plots showing the normalized cross-sectional area of mutants screened in nine-day-old roots. Data from multiple independent experiments were normalized to the average cross-sectional area of the WT control within each experiment. For each genotype, statistical analysis was performed by comparing the pooled normalized cross-sectional area of each mutant to the pooled WT. Differences were tested via one-way analysis of variance (ANOVA) with Dunnett’s multiple comparisons test (*P < 0.05; **P < 0.01), using the WT as controls. *n* indicates the number of individual roots used in the analysis. Representative mutant plastic sections and the WT from the same experiment are shown in Extended Data Fig. 5. The quotation marks indicate that the mutation does not cause a reading frame shift but involves a deletion or insertion in multiples of three nucleotides. **b**, Plastic cross sections and quantification of cross-sectional area, cell number in cross sections and average cross-sectional cell area in the WT, *pll18* *pll19* *pll22* *pll26* and *lbd3* *lbd4* *lbd11* at nine days old. The green boxes highlight the xylem–phloem (x–p) touching phenotype in mutants, a pattern rarely observed in the WT, suggesting vascular cambium cell differentiation in these regions. The cells and area inside the dashed lines were considered in the quantifications of cell number in cross sections and cross-sectional area, respectively. Scale bars, 20 µm. **c**, Comparison of cross-sectional area in *pll* triple and quadruple mutants to the WT in 12-day-old roots. *n* and the grey dots within the box plots represent individual roots used in each analysis (**b**,**c**). Significant differences, indicated by different letters, were determined at an *α* level of 0.05 using a one-way ANOVA with Tukey’s honestly significant difference post-test (**b**,**c**). The data in **a**–**c** are presented as box plots showing the median (centre line), the IQR (box boundaries; 25th and 75th percentiles) and whiskers extending to the minimum and maximum values within 1.5 × IQR of the quartiles. **d**, Frequency of the x–p touching phenotype, where phloem cells are adjacent to xylem vessels, in nine-day-old roots. The x–p touching frequency was calculated as the number of x–p touching events divided by the total number of observed events. *n* indicates the total number of observed events. Source data

**Extended Data Fig. 5:**
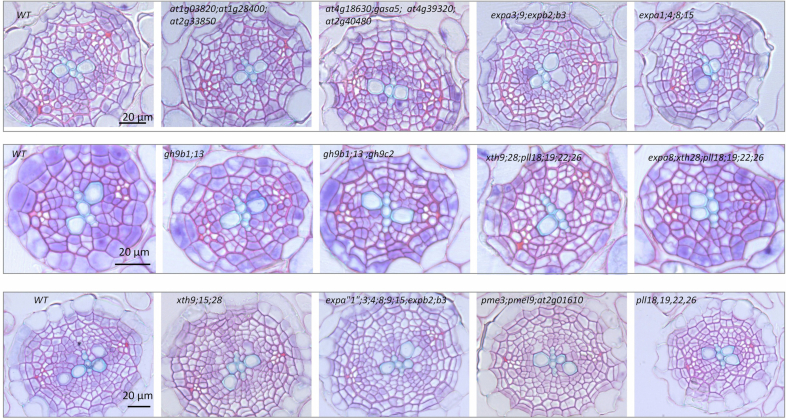
Plastic cross-sections of various cell wall mutants at 9 days old whose quantifications are shown in Fig. 2a. Sections from the same experiment are marked with a gray box. *WT* is included as a reference in each independent experiment.

**Extended Data Fig. 6:**
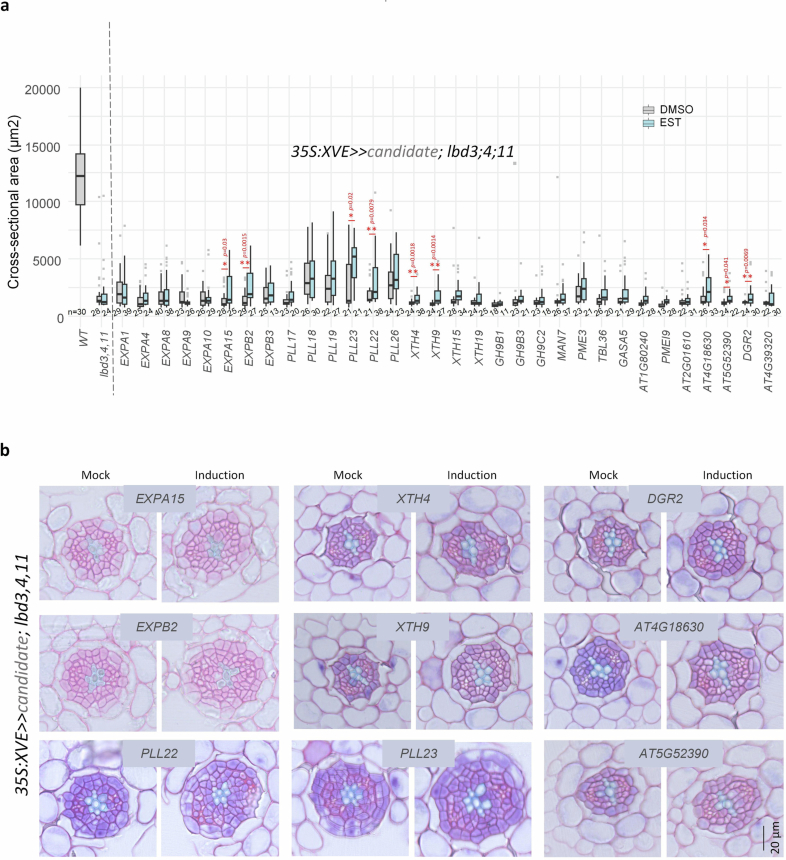
Inducible overexpression of candidate genes in the *lbd3;4;11* background. **a**, Quantification of cross-sectional area of inducible overexpression lines in the *lbd3;4;11* background. Each gene was induced for 4 days in 6-day-old seedlings. T1 transgenic plants were analyzed, and the experiment was conducted once. *WT* and *lbd3;4;11* are shown as references. Boxes represent the first (Q1) and third (Q3) quartiles, with the line inside indicating the median. Data are presented as boxplots showing the median (center line), the interquartile range (IQR, box boundaries: 25th and 75th percentiles), and whiskers extending to the minimum and maximum values within 1.5 × IQR of the quartiles. “n” indicates the number of individual roots used for each treatment. Statistical significance was determined using a two-tailed Student’s t-test. ∗*p* < 0.05; ∗∗*p* < 0.01. **b**, Plastic cross-sections of representative lines showing a slight but significant increase in radial growth upon induction in the *lbd3;4;11* background. Source data

By comparing radial growth, we observed that mutants related to expansins—recognized as the best-characterized cell-wall-loosening agents to date^2^—did not exhibit compromised root radial growth, even when six or seven members of the family were mutated. Unexpectedly, octuple mutants displayed a slight but significant increase in radial growth (Fig. 2a and Extended Data Fig. 5). XTH mutants showed root radial growth comparable to that of the WT. While GLYCOSIDE HYDROLASE mutants exhibited significantly reduced radial growth, their phenotypes were relatively mild. These findings suggest that expansins, XTHs and GLYCOSIDE HYDROLASEs may not be the primary targets of CALBDs in regulating radial growth, or more likely, they may function in a highly redundant manner. Supporting the latter possibility, we observed that conditional overexpression of *expansin*s *A15* and *B2*, as well as *XTH*s *4* and *9*, can significantly (although mildly) suppress the radial growth defects of *calbd* mutants (Extended Data Fig. 6). We also found that 20 more expansins and 11 more *XTH*s are transcribed in secondary tissues (with RNA-seq read counts greater than 50 in one of the five-, six- or nine-day-old WT samples). Among the candidates with unknown functions and strongly CALBD-associated expression profiles (*AT1G03820*, *AT1G28400*, *AT2G33850*, *AT2G40480*, *AT4G18630*, *AT4G39320* and *AT5G52390*), the corresponding mutants exhibited no noticeable radial growth phenotypes (Fig. 2a). We then focused on genes involved in pectin metabolism. Mutants for genes encoding PME3 and PMEIs (*PMEI9* and *AT2G01610*), which potentially regulate the esterification status of homogalacturonan (HG)-type pectin in the cell wall, did not show any defects in root radial growth (Fig. 2a). Finally, we analysed mutations in genes encoding PLLs, which putatively catalyse the cleavage of de-esterified HGs. The quadruple PLL mutant, *pll18* *pll19* *pll22* *pll26*, displayed a notable and significant reduction in radial growth (Fig. 2a). Taken together, with the CRISPR–Cas9-aided mutant screening, we identified four *PLL* genes as major downstream targets of CALBDs in radial growth regulation.

### PLLs regulate radial growth downstream of CALBDs

**Extended Data Fig. 7:**
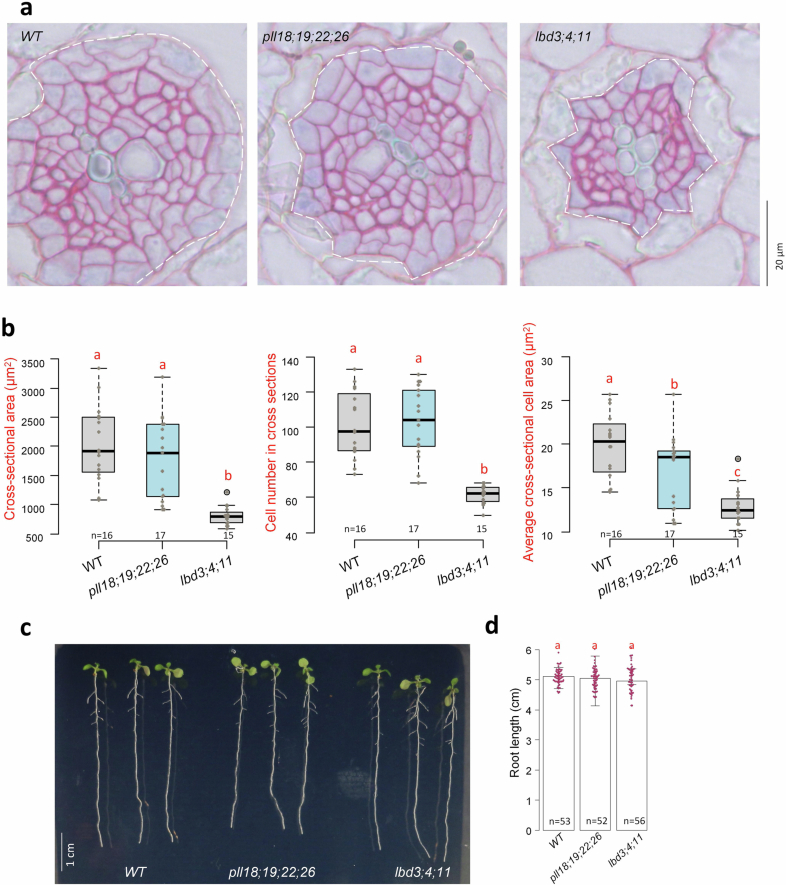
Sections and quantifications of 7-day-old root. **a**. Plastic cross sections of the roots from *WT*, *pll18;19;22;26*, and *lbd3;4;11* at 7 days old. Dashed lines mark the region used for quantification in panel **b**. **b**. Quantification of cross-sectional area, cell number in cross section, and average cross-sectional cell area in *WT*, *pll18;19;22;26* and *lbd3;4;11* at 7 days old. Boxes represent the first (Q1) and third (Q3) quartiles, with the line inside indicating the median. Data are presented as boxplots showing the median (center line), the interquartile range (IQR, box boundaries: 25th and 75th percentiles), and whiskers extending to the minimum and maximum values within 1.5 × IQR of the quartiles. **c**. Whole seedling images of *WT*, *pll18;19;22;26*, and *lbd3;4;11* on the agar plate at 7 days old. Statistical significance was determined using a two-tailed Student’s t-test. **d**. Root length quantifications for the experiments presented in panel **c**. Data are presented as mean ± SD. Significant differences, indicated by different letters, were determined at α = 0.05 using one-way ANOVA followed by Tukey’s HSD test (**b**) or Tamhane’s T2 test (**d**). n and dots within the box plots represent individual roots used in each analysis (**b, d**). Source data

Further characterization of radial growth performance in the *pll18* *pll19* *pll22* *pll26* mutant revealed that, in nine-day-old roots, the reduction in radial growth was due to both decreased numbers of cells in cross sections and reduced radial cell growth, a phenotype similar to that of the *lbd3* *lbd4* *lbd11* mutant (Fig. 2b). In seven-day-old roots, when radial growth was just initiated, the average cell area was already significantly smaller than that in the WT, whereas cell number remained comparable to the WT (Extended Data Fig. 7a,b). These results suggest that the reduction of cell growth in the early developmental stage may restrict cell proliferation in the later stages. Interestingly, we also observed frequent differentiation of vascular cambial stem cells located between the primary xylem and phloem (the xylem–phloem touching phenotype) in *pll18* *pll19* *pll22* *pll26* (Fig. 2b), similar to the phenotype of *lbd3* *lbd4* *lbd11* (ref. ^17^). This observation suggests that PLL-mediated pectin metabolism plays essential roles in cambial stem cell maintenance. These four PLL genes seem to function mainly during root secondary growth, as the primary growth and overall shoot size of their mutants are similar to those of the WT (Extended Data Fig. 7c,d), similar to the observations in the *calbd* mutants^17^ (Extended Data Fig. 7c,d). Additionally, the *pll19* *pll22* *pll26* triple mutant displayed a secondary growth defect similar to that of the *pll18* *pll19* *pll22* *pll26* quadruple mutant, although the frequency of the xylem–phloem touching phenotype was lower (Fig. 2b,c). This finding indicates that *PLL19*, *PLL22* and *PLL26* are the major PLLs in regulating radial growth.

**Fig. 3:**
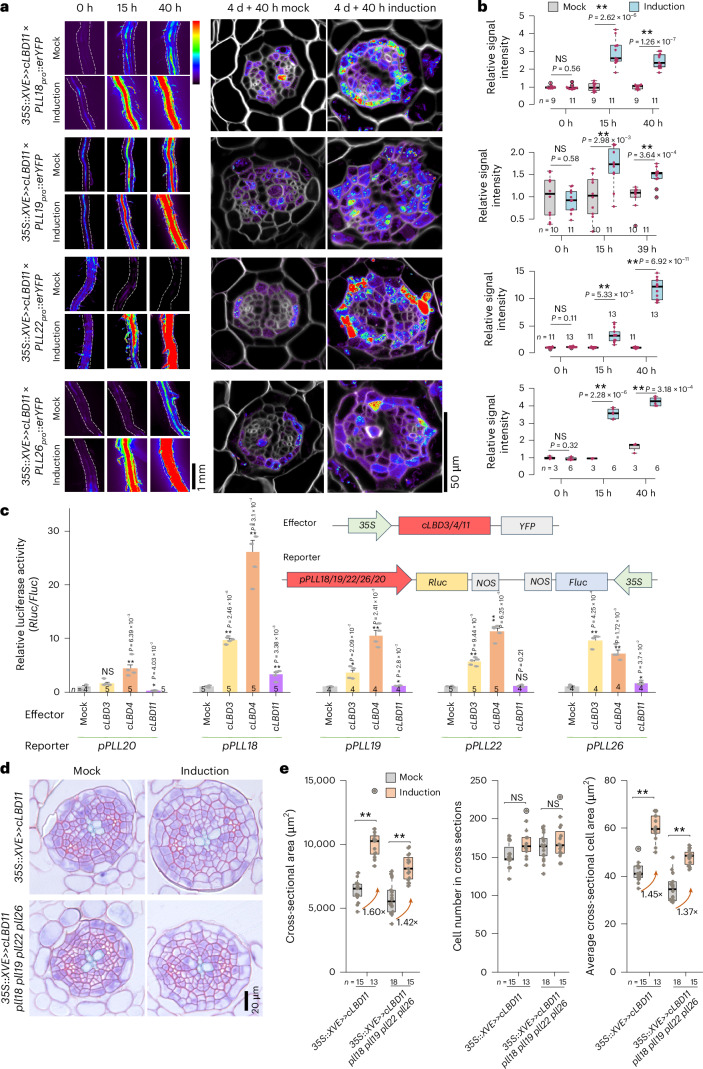
*CALBD*s promote radial growth partially through *PLL* genes. **a**, Stereo microscopy of *PLL* fluorescent reporter lines crossed with the *LBD11* inducible overexpression line. Time-course visualization (left) was performed after 5 μM 17-β-oestradiol treatment or mock treatment in four-day-old roots. Confocal microscopy of cross sections (right) was performed after 40 hours of induction. The dashed lines indicate the root boundaries. The heat map bar represents fluorescence intensity, where purple indicates low intensity and red indicates high intensity. **b**, Quantification of fluorescence intensity in each *PLL* reporter line crossed with the *LBD11* inducible line after the time-course induction from the experiment in **a**. The average signal intensity within the root boundaries was measured, and all signal intensities were normalized to that of the 0-hour mock treatment. **c**, Dual-luciferase transient expression assay in *N. benthamiana* leaves. Effector constructs carried *CALBD* genes driven by the *CaMV 35S* promoter. Reporter constructs contained *PLL* gene promoters driving *Renilla* luciferase (*Rluc*), with firefly luciferase (*Fluc*) under the *CaMV 35S* promoter serving as an internal control. The ratio of *Rluc* to *Fluc* activity was calculated for each sample and normalized to the mock control. The data are shown as mean ± s.d. *n* indicates the number of biological replicates. **d**, Phenotypic analysis following *LBD11* overexpression in either the WT or *pll18* *pll19* *pll22* *pll26* mutant background. A one-day induction was applied to seven-day-old seedlings. **e**, Quantifications of cross-sectional area, cell number in cross sections and average cross-sectional cell area from the experiment in **c**. The orange arrows with numbers indicate the fold increase in cross-sectional area and average cross-sectional cell area after induction compared with the mock treatment. *n* and the dots within the box plots represent individual roots used in each analysis (**b**,**e**). The data in **b**,**e** are presented as box plots showing the median (centre line), the IQR (box boundaries; 25th and 75th percentiles) and whiskers extending to the minimum and maximum values within 1.5 × IQR of the quartiles. Statistical significance was determined using a two-tailed Student’s *t*-test (**b**,**c**,**e**). *P < 0.05; **P < 0.01. A two-way ANOVA showed a significant interaction between genotype (WT versus *pll18* *pll19* *pll22* *pll26*) and *LBD11* overexpression for cross-sectional area (*F* = 5.540, *P* = 0.022) and average cross-sectional cell area (*F* = 5.678, *P* = 0.021), but not cell number (*F* = 0.782, *P* = 0.380). Source data

**Fig. 4:**
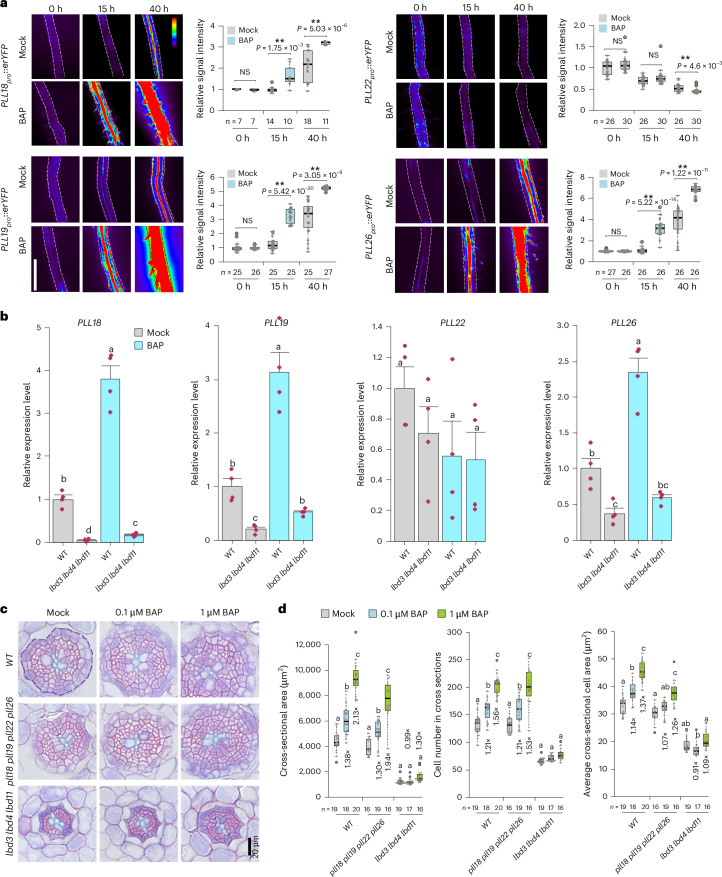
Cytokinin regulates *PLL*s to promote root radial growth in part through *CALBD*s*.* **a**, Time-course visualization and signal intensity quantification of *PLL* fluorescent reporter lines treated with 1 μM 6-benzylaminopurine (BAP) or mock for 0 h, 15 h and 40 h in four-day-old roots. The dashed lines indicate the root boundaries. The average signal intensity within the root boundaries was measured, and all signal intensities were normalized to that of the 0-hour mock treatment. The heat map bar shows fluorescence intensity, where purple indicates low intensity and red indicates high intensity. Statistical significance was determined using a two-tailed Student’s *t*-test. **b**, RT-qPCR analysis of four *PLL* gene expression levels in the WT and *lbd3* *lbd4* *lbd11* backgrounds treated with mock or 1 µM BAP. A one-day treatment was applied to eight-day-old plants, and the main root (0.5–1.5 cm below the hypocotyl–root junction) was collected for analysis. The data are presented as mean ± standard deviation from four biological replicates. A one-way ANOVA with either Tukey’s post hoc test (for equal homogeneous variance) or Tamhane’s post-test (for unequal variance) was applied. **c**, Plastic cross sections of the WT, *pll18* *pll19* *pll22* *pll26* and *lbd3* *lbd4* *lbd11*, treated with mock or BAP (0.1 µM or 1 µM) for two days at six days old. **d**, Quantifications of cross-sectional area, cell number in cross sections and average cross-sectional cell area from the experiment in **c**. *n* and the dots within the box plots represent individual roots used in each analysis (**a**,**d**). A two-way ANOVA was conducted to examine the effects of genotype and treatment, as well as their interaction effect on cross-sectional area, cell number in cross section and average cross-sectional cell area. Post-hoc pairwise comparisons with Bonferroni adjustment were performed to isolate significant differences between individual treatment groups within each genotype. Significant differences, indicated by different letters, were determined at an *α* level of 0.05. The data in **a**,**d** are presented as box plots showing the median (centre line), the IQR (box boundaries; 25th and 75th percentiles) and whiskers extending to the minimum and maximum values within 1.5 × IQR of the quartiles. Each point represents an individual data point from root images (**a**), biological replicates (**b**) or root sections (**d**). Source data

**Extended Data Fig. 8:**
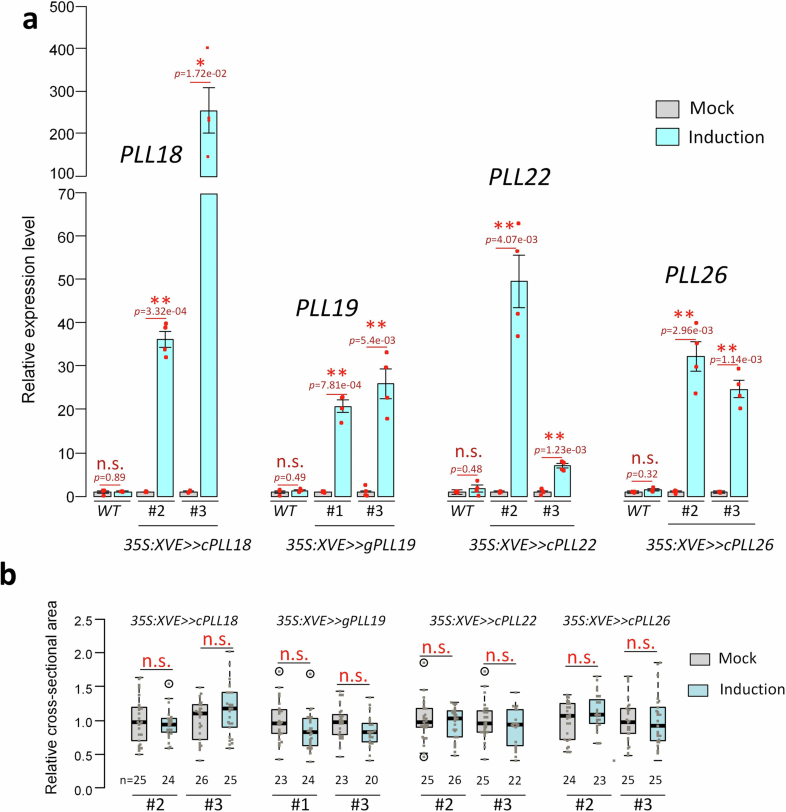
Overexpression of *PLL* genes in the *WT* background. **a**, RT-qPCR analysis of the expression levels of four *PLL* genes in their respective overexpression lines. Four-day-old seedlings were treated with 5 μM estradiol for six days, and whole roots were used for RNA extraction. Data are presented as mean ± standard deviation from four biological replicates. Two independent lines were analyzed for each construct. **b**, Quantification of the relative cross-sectional area in four *PLL* overexpression lines in the *WT* background. Transverse root sections were taken 0.5 cm below the root–hypocotyl junction. The cross-sectional area of each line was normalized to its corresponding mock control. Statistical significance was assessed using a two-tailed Student’s t-test. n.s., not significant; *, *p* < 0.05; **, *p* < 0.01. Boxes (b, d) represent the first (Q1) and third (Q3) quartiles, with the line inside indicating the median. Data are presented as boxplots showing the median (center line), the interquartile range (IQR, box boundaries: 25th and 75th percentiles), and whiskers extending to the minimum and maximum values within 1.5 × IQR of the quartiles. “n” indicates the number of individual roots used for each analysis. Source data

To assess whether *PLL* overexpression is sufficient to promote secondary growth, we induced the expression of *PLL18*, *PLL19*, *PLL22* and *PLL26* in the WT background during secondary growth. Although reverse transcription quantitative real-time PCR (RT-qPCR) confirmed elevated transcript levels upon induction, *PLL* overexpression lines did not show a significant increase in total root secondary tissue area (Extended Data Fig. 8). This result suggests that PLL function requires precise spatio-temporal regulation or additional cofactors for its full activity. To validate that these PLLs act downstream of CALBDs, we first generated transcriptional reporters of them. Expression analysis revealed their broad expression in the root secondary tissue. The induction of *LBD11* strongly and rapidly induced their expression (Fig. 3a,b). Conversely, the expression of *PLL18*, *PLL19* and *PLL26* is significantly downregulated in the *lbd3* *lbd4* *lbd11* mutant (Fig. 4b), while the expression of *PLL22* is only slightly downregulated. These experiments thus confirmed our RNA-seq profiling data. To assess whether *CALBD*s directly activate these *PLL*s, we conducted a dual-luciferase transient expression assay in *Nicotiana benthamiana*. *PLL20*, which was not upregulated by *LBD3* or *LBD11* in RNA-seq, served as a negative control and showed no activation by *LBD3* or *LBD11*. In contrast, all other tested promoters—*PLL18*, *PLL19*, *PLL22* and *PLL26*—were significantly activated by *CALBD*s, except for *PLL22*, which was not significantly activated by *LBD11* (Fig. 3c). Additionally, the LBD3,4 binding motif^21^ 5′-(G)CGGC(G)-3′ was detected within 2 kb upstream of *PLL18*, *PLL19* and *PLL22* (Supplementary Table 8), further supporting direct transcriptional activation.

To investigate whether CALBDs function through PLLs, we crossed the *LBD11* inducible overexpression line with the *pll18* *pll19* *pll22* *pll26* mutant. The *pll18* *pll19* *pll22* *pll26* mutant exhibited partial resistance to *LBD11* overexpression: *LBD11* induction led to a larger fold change in cell growth and whole radial growth in the WT than in the *pll18* *pll19* *pll22* *pll26* background (Fig. 3d,e). Furthermore, overexpression of *PLL22* partially rescued the radial growth defect of the *lbd3* *lbd4* *lbd11* mutant (Extended Data Fig. 6). Together, these data demonstrate that PLLs function downstream of CALBDs in regulating radial growth. We also noted that the radial growth defect in *pll18* *pll19* *pll22* *pll26* is not as strong as that in *calbd* mutants, and *pll18* *pll19* *pll22* *pll26* remains substantially responsive to *LBD11* overexpression. Considering the large number of factors transcriptionally regulated by CALBDs, it is likely that the regulation of radial growth involves a concerted action of multiple cell-wall-related agents alongside these four PLLs. In summary, CALBDs regulate radial growth and cambium stem cell maintenance in part through PLLs, with additional contributions from other cell-wall-modifying agents.

### A cytokinin–CALBD–PLL regulatory module controls radial growth

Since CALBDs act downstream of cytokinin in regulating radial growth^17^, we then investigated whether a cytokinin–CALBD–PLL pathway exists in the regulation of root radial growth. Among *PLL18*, *PLL19*, *PLL22* and *PLL26*, we found that the expression of *PLL18*, *PLL19* and *PLL26* can be induced by cytokinin treatment, as shown via reporter analysis (Fig. 4a). However, *PLL22* showed rapid induction only by *LBD11* (Figs. 3a,b and 4a). To further explore the role of CALBDs in cytokinin-mediated *PLL* expression, we examined the expression of all four *PLLs* in the *lbd3* *lbd4* *lbd11* mutant upon cytokinin treatment using RT-qPCR. We found that *PLL18*, *PLL19* and *PLL26* expression levels were significantly less induced by cytokinin in the *lbd3* *lbd4* *lbd11* mutant than in the WT background, although they were still induced to some extent (Fig. 4b). This suggests that cytokinin regulates the expression of *PLL18*, *PLL19* and *PLL26* primarily through CALBDs. Next, we treated *pll18* *pll19* *pll22* *pll26* and *lbd3* *lbd4* *lbd11* with cytokinins and compared their responses to that of the WT. We found that in response to cytokinin treatment, *pll18* *pll19* *pll22* *pll26* displayed lower fold changes in terms of cross-sectional area and average cross-sectional cell area than WT roots, while the fold change in the number of cells in cross sections was comparable to that of the WT. Cell growth in *pll18* *pll19* *pll22* *pll26* was resistant to low levels of applied cytokinins (0.1 μM) (Fig. 4c,d). Although the *pll18* *pll19* *pll22* *pll26* mutant showed resistance to cytokinin-induced radial growth, it was not as resistant as the *lbd3* *lbd4* *lbd11* mutant (Fig. 4c,d). These findings indicate that cytokinin promotes radial growth at least partially through PLLs. In conclusion, our findings revealed a cytokinin–CALBD–PLL pathway involved in the regulation of radial growth.

### CALBDs and PLLs modulate primary cell wall chemical and mechanical properties

**Fig. 5:**
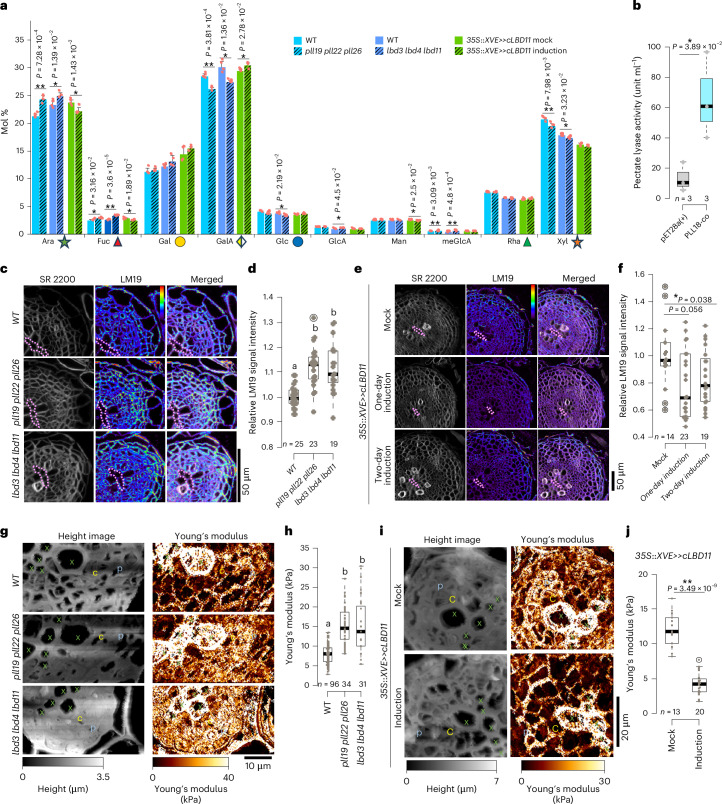
*CALBD*s and *PLL* genes regulate cell wall remodelling during root radial growth. **a**, Mole percent (Mol %) of cell wall monosaccharide composition in 12-day-old mutants and *LBD11* inducible lines after two days of induction. Glc, glucose; GlcA, glucuronic acid; meGlcA, 4-*O*-methylglucuronic acid. Different shapes and colours represent the monosaccharides from the schematic diagram in Extended Data Fig. 9, which simplistically depicts the structures of major polysaccharides in a growing cell. The data are presented as mean ± standard deviation from four biological replicates. Monosaccharides in the mutant were compared to those in the WT in the same experimental round (filled with the same colour). Monosaccharides from the *LBD11* inducible line were compared between 5 μM 17-β-oestradiol treatment and mock treatment following a two-day treatment in ten-day-old roots. Statistical significance was determined using a two-tailed Student’s *t*-test. *P < 0.05; **P < 0.01. **b**, Pectate lyase activity of PLL18-co compared with the vector control (pET28a(+)). Statistical significance was determined using a two-tailed Student’s *t*-test based on three biological replicates. **c**, Immunofluorescence detection of unesterified HG using the LM19 monoclonal antibody in ten-day-old WT, *pll19* *pll22* *pll26* and *lbd3* *lbd4* *lbd11* mutant roots. The dashed lines indicate the primary xylem axis in the sections (**c**,**e**). SR2200 signal for cell wall (greyscale) is shown on the left, LM19 signal (heat map) in the middle and the merged image on the right (**c**,**e**). The heat map bar shows signal intensity, where purple indicates low intensity and red indicates high intensity (**c**,**e**). **d**, Quantification of immunofluorescence signal intensity from the experiment shown in **c**. The immunofluorescence signal intensity in the cell wall region was first extracted, and then all values were normalized to the mean signal intensity of the WT. *n* and the grey dots within the box plots represent individual sections analysed for each genotype. Significant differences, indicated by different letters, were determined at an *α* level of 0.05 using a one-way ANOVA with Tamhane’s post-test. **e**, Immunofluorescence detection of unesterified HG using the LM19 monoclonal antibody in *LBD11* inducible overexpression lines. Roots were analysed after one or two days of induction in ten-day-old plants, and a two-day mock treatment in ten-day-old roots was used as the control. **f**, Quantification of immunofluorescence signal intensity from the experiment shown in **e**. The immunofluorescence signal intensity in the cell wall region was first extracted, and then all values were normalized to the mean signal intensity of the mock treatment. *n* and the grey dots within the box plots represent individual sections analysed for each genotype. Statistical significance was determined using a two-tailed Student’s *t*-test. **g**, Mechanical property measurements using AFM in ten-day-old WT, *pll19* *pll22* *pll26* and *lbd3* *lbd4* *lbd11* plants. Height images (left) and Young’s modulus maps (right) are shown. p, phloem cell; x, xylem vessel; c, vascular cambium. ScanAsyst-Fluid probe was used for the experiment. **h**, Quantification of Young’s modulus in the cambium of WT, *pll19* *pll22* *pll26* and *lbd3* *lbd4* *lbd11* plants from the experiment shown in **g**. Additional quantification of Young’s modulus across xylem, cambium and phloem is presented in Supplementary Fig. 2a,b. This quantification was conducted by calculating the average stiffness in a 3 µm × 3 µm area of the cell wall region. *n* and the grey dots in the box plots represent individual measurements from the cell wall region of the corresponding cell type. Significant differences, indicated by different letters, were determined at an *α* level of 0.05 using a one-way ANOVA with Tamhane’s post-test. **i**, AFM-based mechanical property measurements in the *LBD11* inducible overexpression line. A one-day induction or mock treatment was applied to seven-day-old plants. Height images and Young’s modulus maps are shown. **j**, Quantification of Young’s modulus in the cambium region of the *LBD11* inducible overexpression line from the experiment in **i**. Quantification of Young’s modulus across the xylem, cambium and phloem is shown in Supplementary Fig. 2c. Statistical significance was determined using a two-tailed Mann–Whitney test. *P < 0.05; **P < 0.01. *n* and the grey dots in the box plots represent individual measurements from the cell wall region of the corresponding cell type. The data in **b**,**d**,**f**,**h**,**j** are presented as box plots showing the median (centre line), the IQR (box boundaries; 25th and 75th percentiles) and whiskers extending to the minimum and maximum values within 1.5 × IQR of the quartiles. Source data

**Extended Data Fig. 9:**
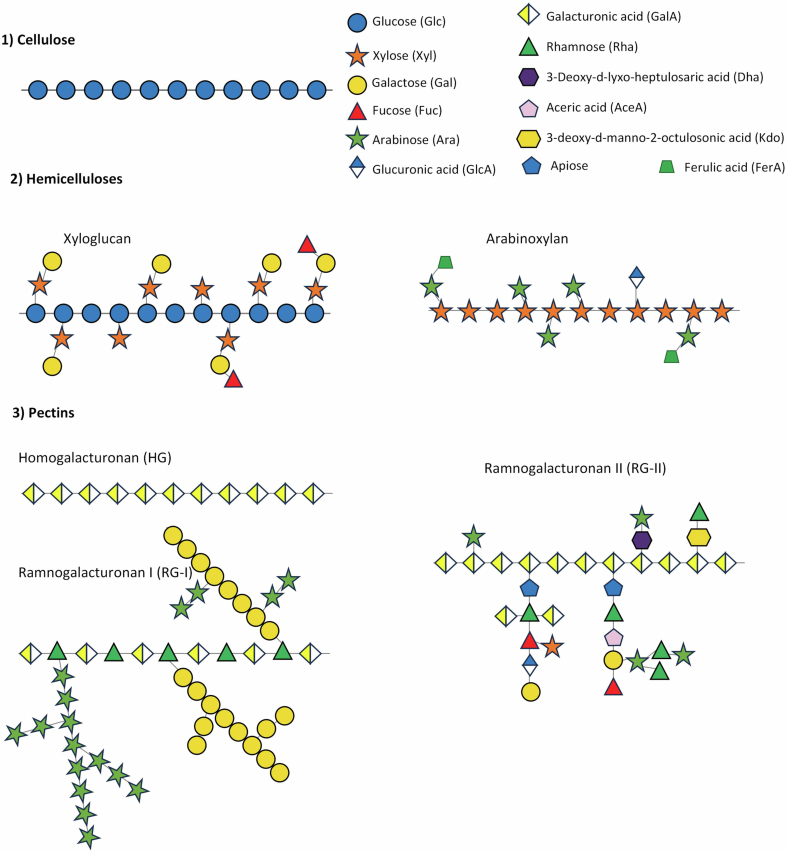
A simplified schematic diagram illustrating the structure of the dominant polysaccharides in a growing cell. The primary wall is mainly composed of cellulose, hemicellulose, and pectin. Pectins, rich in galacturonic acid. The two most abundant pectins are homogalacturonan (HG) and rhamnogalacturonan-I (RG-I). HG consists of linear galacturonic acid chains, while RG-I has an alternating rhamnose–galacturonic acid backbone decorated with arabinose and galactose side chains. Figure adapted with permission from ref. ^1^, Springer Nature Limited.

We then explored the cell wall basis underlying radial growth regulation by CALBDs and PLLs. To assess changes in cell wall polysaccharide composition, we performed a cell wall monosaccharide composition analysis using trimethylsilyl derivatization. This analysis was conducted on samples isolated from 12-day-old roots of the WT, *lbd3* *lbd4* *lbd11*, *pll19* *pll22* *pll26* and the *LBD11* conditional overexpression line (two days of induction or mock). We found that, compared with the WT, both *pll19* *pll22* *pll26* and *lbd3* *lbd4* *lbd11* mutants showed similar changes in tested monosaccharide compositions (Fig. 5a). Specifically, we observed significant increases in arabinose (Ara), fucose (Fuc) and 4-*O*-methylglucuronic acid, alongside decreases in galacturonic acid (GalA) and xylose (Xyl). No significant changes were observed for mannose (Man), galactose (Gal) or rhamnose (Rha). After a two-day induction of *LBD11*, we detected an opposite pattern, with decreases in Ara and Fuc and increases in GalA. CALBDs and PLLs therefore have similar effects on cell wall polysaccharide composition in secondary tissues of *Arabidopsis* roots, indicating that CALBDs regulate cell wall composition through PLLs. Since GalA is present mainly in HG-type pectins (Extended Data Fig. 9) and Ara and Fuc exist also in other pectin types^19^, the consistent changes of these three monosaccharides in *pll19* *pll22* *pll26* and *lbd3* *lbd4* *lbd11* mutants, coupled with the opposite change in the *LBD11* overexpression line, indicate that CALBDs and PLLs primarily regulate pectin metabolism in the primary cell walls of secondary tissues.

**Extended Data Fig. 10:**
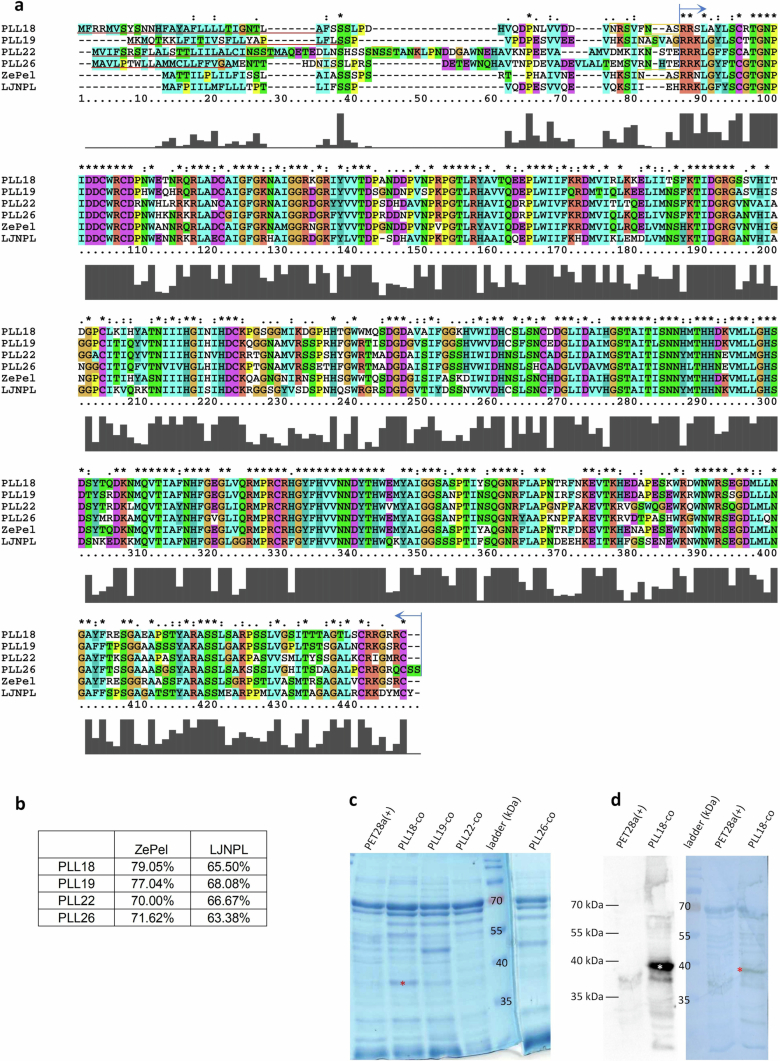
Sequence alignment, expression, and purification of *Arabidopsis* pectate lyases. **a**, Alignment of pectate lyase protein sequences. *Arabidopsis* PLL18, PLL19, PLL22, and PLL26 were aligned with biochemically characterized pectate lyases from *Zinnia elegans*^22^ (*ZePel*) and *Lotus japonicus*^23^ (*LJNPL*) using CLUSTALX 2.1. Residues fully conserved across all six sequences are marked with asterisks (*), while conserved and semi-conserved residues are indicated by colons (:) and periods (.), respectively. Predicted signal peptides are underlined, potential glycosylation sites are marked with box and the regions cloned for enzymatic activity assays are indicated by arrows. **b**, Pairwise sequence similarity between *Arabidopsis* PLLs and ZePel and LJNPL proteins. **c**, SDS-PAGE analysis of pectate lyases expressed from pET28a(+) constructs and purified via Ni-NTA affinity chromatography. Asterisk (*) indicates the recombinant PLL18-co protein. **d**, Western blot confirmation of the purified recombinant PLL18-co protein using anti-6×His antibody. Shown are the Western blot (left) and the corresponding amido black-stained membrane (right). The Western blot image was captured using an iBright 1500 (Invitrogen) with an exposure time of 0.5 second. This experiment was conducted in three biological replicates (**c, d**). Source data

Since *Arabidopsis* PLLs are annotated as putative pectate lyases and no functional *Arabidopsis* pectate lyase has been biochemically validated to date, we compared the sequences of PLL18, PLL19, PLL22 and PLL26 with functionally characterized pectate lyases from *Zinnia elegans* (Ze*Pel*)^22^ and *Lotus japonicus* (Lj*NPL*)^23^ (Extended Data Fig. 10a). All four PLLs showed high sequence similarity with functional Ze*Pel* and Lj*NPL* (Extended Data Fig. 10b). After failing to express the *Arabidopsis* PLLs in *Escherichia coli*, we considered the potential impact of codon usage bias in *E. coli*^24,25^ and generated codon-optimized (co) versions of each PLL, fused to a carboxy-terminal His tag. Among them, PLL18-co exhibited the highest expression (Extended Data Fig. 10c). Despite the presence of some non-specific background bands after purification (Extended Data Fig. 10c), an ~40-kDa band corresponding to PLL18-co was detected and confirmed via western blot using an anti-His antibody (Extended Data Fig. 10d). The purified PLL18-co protein exhibited significantly higher pectate lyase activity than the empty vector control (pET28a(+)) (Fig. 5b), indicating that PLL18-co is a functional pectate lyase in *Arabidopsis*.

These four PLLs are predicted to degrade de-esterified HGs; we therefore used LM19, an antibody that labels de-esterified HGs^26^, to detect HG de-esterification levels in the cell walls of *calbd* and *pll* mutants, as well as the *LBD11* inducible overexpression line. The signal intensity of LM19 significantly increased in the primary cell walls of secondary tissues of both *pll19* *pll22* *pll26* and *lbd3* *lbd4* *lbd11* mutant roots, compared with the WT (Fig. 5c,d). Compared with the mock treatment, we observed a decreased signal intensity after a two-day *LBD11* induction (Fig. 5e,f). Our results therefore showed that both CALBDs and PLLs regulate de-methylesterification levels of HGs in the primary cell walls of secondary tissues of *Arabidopsis* roots.

In different contexts, both high and low levels of de-methylesterification levels of HGs are associated with changes in cell wall stiffness^27^. To directly assess cell wall mechanical properties in root secondary tissue, we performed atomic force microscopy (AFM) analysis on the WT, *pll19* *pll22* *pll26* and *lbd3* *lbd4* *lbd11*, as well as the *LBD11* inducible overexpression line (Supplementary Fig. 1). We found that in the WT root undergoing radial growth, the cell walls in the meristematic cambium region display higher elasticity (less stiffness) than cell walls in the differentiated xylem and phloem region (Fig. 5g,h and Supplementary Fig. 2a). As expected, xylem vessels with lignified secondary cell walls showed the highest stiffness (Supplementary Fig. 2a). After a one-day induction of *LBD11*, we observed enhanced cell wall softening (increased elasticity/decreased stiffness) in the whole secondary tissue, while in the *pll19* *pll22* *pll26* and *lbd3* *lbd4* *lbd11* mutants, stiffer cell walls were observed (Fig. 5g–j and Supplementary Fig. 2b,c). Since both mutants show consistent changes in de-methylesterified HG levels as indicated by LM19 labelling, we assume that the enhanced de-methylesterified HG levels may explain their increased cell wall stiffness. In summary, CALBDs and PLLs determine the primary cell wall properties at the chemical and physical levels by acting on pectin metabolism, which may underlie the reduced cell growth and cell proliferation in both mutants.

## Discussion

Cell wall constraints and turgor pressure are the primary mechanical factors controlling plant cell growth^28^. In this study, we focused on cell-wall-agent-mediated cell growth during radial growth of *Arabidopsis* roots. The candidate cell-wall-loosening agents, including expansins, XTHs and pectin-modifying enzymes, function in various developmental and physiological processes^2,6,29^. However, with respect to cell growth regulation itself, their genetic importance and impact on cell wall composition have been reported mostly in a few highly specialized plant tissues, such as the pollen tube and root hairs^30–32^. Despite the central role of vascular tissues in vascular plants, the molecular regulation of vascular cell growth remains largely unexplored. In this study, we systematically investigated the roles of these candidate cell-wall-loosening agents in the radial growth of *Arabidopsis* roots using a reverse genetic screening approach. Expansins are widely recognized as key players in cell growth, but their biological role has been explored mainly via overexpression studies^3^, probably due to heavy genetic redundancy. In this study, we generated high-order mutants of expansins and observed no compromised root radial growth. We therefore conclude that expansins may have minor roles in radial growth control, or alternatively, they regulate radial cell growth in a highly redundant manner. We noticed that overexpression of expansins partially rescued the radial growth defect of the *lbd3* *lbd4* *lbd11* mutant (Extended Data Fig. 6), thus supporting the latter option. A similarly redundant and promotive role in radial cell growth was also observed for XTHs (Fig. 2a and Extended Data Fig. 6).

We identified a set of *PLL* genes (*PLL18*, *PLL19*, *PLL22* and *PLL26*) to function downstream of cytokinins and CALBDs in regulating *Arabidopsis* root radial growth. Similar to the *calbd* mutant, *pll* mutants exhibit reduced radial cell growth and proliferation, resulting in a defect in radial growth of secondary tissue of *Arabidopsis* root (Fig. 2b and Extended Data Fig. 7a,b). We also observed frequent differentiation of vascular cambial stem cells in the *pll* mutants, similar to the *calbd* mutant^17^ (Fig. 2b), suggesting that PLL-mediated pectin metabolism is essential for cambial stem cell maintenance. However, the phenotype in *pll* mutants is milder than that in the *calbd* mutants (Fig. 2b). Given that our study showed CALBDs broadly regulate cell-wall-related genes and metabolic processes, it is likely that CALBDs control cell growth and proliferation through coordinated regulation of multiple cell wall agents. Alternatively, CALBDs primarily regulate cell growth, which in turn leads to changes in cell wall composition. Given that our RNA-seq datasets of *calbd* mutants and CALBD inducible overexpression lines—before the appearance of severe growth defects—are enriched with genes associated with cell wall modification, the first scenario appears more likely. In our study, we established a cytokinin–CALBD–PLL regulatory module that governs *Arabidopsis* root radial growth. Cytokinin promotes *PLL18*, *PLL19* and *PLL26* transcription largely in an LBD-dependent manner, and its promoting effect on radial growth appears to be mediated, at least in part, by *PLL18*, *PLL19*, *PLL22* and *PLL26* (Figs. 3 and 4). Our findings thus provide a foundation for further investigation into hormone-mediated regulation of plant growth and cell wall metabolism.

Although pectate lyase activity has been demonstrated for PLLs from *Z. elegans*^22^ and *L. japonicus*^23^, the enzymatic activity of *Arabidopsis* PLLs has yet to be experimentally confirmed. We attempted to express *Arabidopsis* PLL19, PLL22 and PLL26 with His- or GST-tags in *E. coli*, but these efforts failed to yield soluble protein. Similar difficulties have been reported by Vogel et al.^33^ and Kalmbach et al.^12^ for PLL13 and PLL12 in *E. coli* or *Pichia*. However, since *Arabidopsis* codon usage differs from that of *E. coli*, it is possible that this discrepancy negatively affects the expression and solubility of the recombinant PLL proteins. We therefore synthesized codon-optimized PLL genes, including PLL18, which exhibited relatively high expression levels in *E. coli*. Following one-step purification, we used a commercial assay to demonstrate that PLL18 possesses pectate lyase activity (Fig. 5b and Extended Data Fig. 10). Although the other cambium PLLs failed to express in *E. coli*, considering their protein sequence similarity, it is likely that they also function as pectate lyases. Since our results showed that PLL18 degrades pectate, we then investigated the cell wall basis underlying the radial growth defect observed in the LBD and PLL manipulation lines. A cell wall monosaccharide composition analysis in *calbd* and *pll* mutant roots revealed altered pectin-related monosaccharide levels, which were logically opposite to those observed in the *LBD11* overexpression line (Fig. 5a). Mutations in PLLs, which are putative pectate lyases involved in degrading de-methylesterified HG-type pectins, led to a reduction in pectin-relevant monosaccharide contents (Fig. 5a). This counterintuitive result indicates that the cell wall metabolism is highly dynamic and thus complex. Supporting this, constitutive overexpression of α-expansin resulted in growth inhibition and cell wall stiffening in both *Arabidopsis* and tomato^34,35^. Immunolabelling analysis with LM19 labelling also showed that *calbd* and *pll* mutants had increased levels of HG de-methylesterification (Fig. 5c). De-esterified HGs are associated with enhanced cell wall stiffness in specific developmental contexts. For instance, at the hemispherical apex of growing pollen tubes, de-esterified HG correlates with a more rigid wall structure and the halting of wall expansion^36,37^. AFM analysis further indicated that the *calbd* and *pll* mutants have stiffer cell walls than the WT roots, while *LBD11* overexpression led to cell wall softening. Although we cannot fully exclude the influence of cell size on AFM measurements, these findings indicate that increased levels of HG de-methylesterification reduce cell wall elasticity, thereby restricting radial cell growth and proliferation in the secondary tissue of *Arabidopsis* root. Supporting this, the apical region of the *Arabidopsis* stem with more esterified pectins is softer and faster growing than the lower region of the stem with less esterified pectins^38^. Recent studies have shown that a tomato *LBD* gene, Sl*LOB1*, regulates a wide array of genes associated with cell wall composition during tomato fruit softening^39^. In other studies, silencing the pectate-lyase-encoding gene Sl*PL* (*Solyc03g111690*), a homologue of *Arabidopsis PLL18* and *PLL22*, significantly enhances tomato fruit firmness^40,41^. These findings suggest a conserved function for the LBD–PLL module across different species.

## Methods

### Plant materials and growth conditions

The Col-0 ecotype was used as the WT. The seeds were surface sterilized via sequential incubation in 20% chlorine solution and 70% ethanol for 1 min each, with vortexing, followed by two rinses in sterile water. The sterilized seeds were stratified in darkness at 4 °C for two days before being sown on plates containing 50 ml of half-strength germination medium (1/2 GM). This 1/2 GM consisted of 0.5× Murashige and Skoog salts, 0.8% plant agar, 1% sucrose and 0.5 g l^−1^ MES, adjusted to pH 5.8. The plates were placed vertically in a growth chamber set at 22 °C with a 16-hour light and 8-hour dark cycle. The day the plates were placed in the growth chamber was marked as day 0. For chemical treatments, 17-β-oestradiol was dissolved in dimethyl sulfoxide to prepare a 20 mM stock solution, and a 5 μM working concentration was used. Similarly, BAP was prepared as a 10 mM stock solution in dimethyl sulfoxide, with working concentrations of 0.1 μM or 1 μM. Treatments were performed by transferring seedlings germinated on 1/2 GM to medium supplemented with either 17-β-oestradiol or BAP. Seedlings transferred to medium containing an equivalent volume of dimethyl sulfoxide served as mock controls.

### Cloning and transformation

The promoters of *PLL18*, *PLL19*, *PLL22* and *PLL26*, with respective lengths of 707 bp, 2,671 bp, 2,523 bp and 2,579 bp, upstream of the coding sequence, were amplified and cloned into either the first box donor vector pDONR_1R4z (for *PLL18* and *PLL26*) via BP reaction or the entry vector 1R4z-BsaI-ccdB-BsaI via Golden Gate cloning^42^. For the overexpression assay, either the genomic or coding sequences of the candidate genes were amplified and cloned into second box donor vector pDONR221z via BP reaction or the entry vector 221z-BsaI-ccdB-BsaI via Golden Gate cloning. The 221z-BsaI-ccdB-BsaI entry vector was constructed as previously described^43^. All primers are listed in Supplementary Table 4. The expression constructs were generated by combining the first box promoter or *p1R4-35S*::*XVE*^44^, the second box *p221z-erYFP*^44^ or candidate genes and the third box *p2R3a-nosT*^44^ with the seed coat RFP selection destination vector FRm43GW^43^ via LR reaction. All generated expression constructs are listed in Supplementary Table 5. The reporter constructs *PLL18*_*pro*_::*erYFP*, *PLL19*_*pro*_::*erYFP*, *PLL22*_*pro*_::*erYFP* and *PLL26*_*pro*_::*erYFP* were introduced into the Col-0 background, and the overexpression constructs were introduced into the *lbd3* *lbd4* *lbd11* mutant background. Overexpression lines were analysed in the T_1_ generation. The seeds used in this study are listed in Supplementary Table 6.

### CRISPR–Cas9 screen of candidate genes

A 1,660-bp upstream fragment of the *RPS5A*^20^ coding sequence was cloned into the first box entry vector 1R4z-BsaI-ccdB-BsaI^42^ to generate 1R4z-pPRS5A. The intronized *Cas9* (*iCas9*)^20^ was cloned into the second box entry vector 221z-BsaI-ccdB-BsaI to generate 221z-iCas9. Target sites of the candidate genes were determined using the online genome editing tool CHOPCHOP^45^ and are listed in Supplementary Table 2. For each candidate gene, a single target site was selected. The targets sites were integrated into the single guide RNA (sgRNA) expression cassette via a two-round PCR step, followed by recombination into the third box entry vector 2R3z-BsaI-ccdB-BsaI^43^, as previously described^43^. For each sgRNA expression cassette, the promoter At*U6-26* was amplified from the vector pAGM55261 (Plasmid No. 153210 from Addgene)^20^, and the sgRNA scaffold together with the terminator was amplified from pHEE2E-TRI (Plasmid No. 71288 from Addgene)^46^. One or two sgRNA expression cassettes were combined into the vector 2R3z-BsaI-ccdB-BsaI^43^. LR reactions were performed to combine the resulting entry vectors 1R4z-pPRS5A, 221z-iCas9 and 2R3z-target-sgRNA into the destination vector pFRm43GW with seed coat RFP selection^43^, or the modified seed coat GFP selection vector pFG7m34GW^43^ with kanamycin resistance (pFG7m34GW-Kan), in which the original spectinomycin resistance gene was replaced with a kanamycin resistance gene via Omega PCR as previously described^43^. For multiplex genome editing, we also developed a co-dipping method. Briefly, we first mixed the seed coat RFP selection construct (spectinomycin resistance) with the seed coat GFP selection construct (kanamycin resistance) and then transformed the mixture into the *Agrobacterium* competent cells. The positive transformants were screened on growth medium containing both spectinomycin and kanamycin. The CRISPR constructs were segregated out in the T_2_ generation. All primers are listed in Supplementary Table 4.

### RNA-seq profiling and data analysis

For *35S*::*XVE* ≫ *cLBD4*, RNA-seq profiling was conducted using the same protocol as published for *35S*::*XVE* ≫ *cLBD3* and *35S*::*XVE* ≫ *cLBD11* (ref. ^17^). Briefly, seeds were germinated on 1/2 GM plates for nine days, then transferred to plates with either 5 μM 17-β-oestradiol induction or mock treatment for 8 or 24 hours. The 0.5–2 cm region of the main root below the root–hypocotyl junction, with visible lateral roots removed, was then collected for analysis. For the WT, *lbd3* *lbd4* and *lbd3* *lbd4* *lbd11*, the 0.5–1.5 cm region of the main root (with visible lateral roots removed) below the root–hypocotyl junction was collected from five-, six- and nine-day-old seedlings for analysis, with each sample including three biological replicates. RNA isolation, integrity checks, library preparation and analysis were performed as previously described^17^. Briefly, total RNA was isolated using the GeneJET Plant RNA Purification Kit (Thermo Fisher Scientific). RNA integrity was verified prior to library construction. Sequencing libraries were prepared with the TruSeq Stranded Total RNA Library Prep Kit (Illumina). Data analysis was conducted using Chipster X 2.1 (https://chipster.csc.fi) and RStudio v1.4.1106 (https://www.rstudio.com). Genes with RNA-seq reads greater than ten in any WT sample at five, six or nine days were considered expressed. Significantly differentially expressed genes were identified using a threshold of *P*_adj_ < 0.05, with an additional filter of |log2FC| > 1 applied in some cases to highlight the most significantly changed genes. Differentially expressed genes are listed in Supplementary Table 7. The raw data are available in the Sequence Read Archive under accession number PRJNA1288429.

### Sections and microscopy

Plastic sectioning was conducted as previously described^47^, at a fixed root position 5 mm below the hypocotyl. Sections were sequentially stained with 0.05% (w/v) toluidine blue and 0.05% (w/v) ruthenium red, mounted in water and visualized using a Leica 2500 microscope. For vibratome sectioning, root samples were fixed in 4% paraformaldehyde (PFA, Sigma) prepared in 1× phosphate-buffered saline solution (PBS, pH 7.2) for one hour under vacuuming or overnight at 4 °C. After fixation, the samples were washed twice with 1× PBS. The root samples were then aligned side by side on a glass slide placed on ice and embedded in 4% agarose prepared in 1× PBS. Cross sections of 200 µm thickness were obtained for confocal analysis. Before imaging with a Leica Stellaris 8 confocal microscope (×63 objective), plant cell walls were visualized by incubating sections in staining solution SR2200 (0.1% in 1× PBS). For stereo microscopy, live seedlings on growth plates were imaged directly at the indicated time points using a Leica M165 FC fluorescence stereo microscope.

### Image processing and quantifications

Quantification of cross-sectional area and number of cells in cross section was performed using Fiji ImageJ v1.52 software. Heat maps for fluorescence intensity from stereo and confocal microscopy images were generated using Leica AF Lite Software, while Fiji was used to quantify intensity values across samples. For comparative analysis, microscopy settings were standardized and maintained consistently throughout each experiment to ensure reliable comparisons.

### Dual-luciferase transient expression assay

To perform the dual-luciferase transient expression assay, we first constructed both effector and reporter plasmids. The effector constructs were generated using the MultiSite Gateway system. Each effector consisted of a *CaMV35S* promoter (pEN-L4-35S-L1 (ref. ^48^)), a *CALBD* coding sequence (p221z-cLBD3, p221z-cLBD4 or p221z-cLBD11, all lacking stop codons), and 2R3e-4xgly-venYFP-3AT^44^, cloned into the destination vector pBm43GW^49^.

For the reporter constructs, we used the same promoter regions from the transcriptional reporter lines to drive Rluc expression. These included *pPLL18* (707 bp upstream), *pPLL19* (2,671 bp), *pPLL22* (2,523 bp), *pPLL26* (2,579 bp) and *pPLL20* (1,818 bp), which served as a negative control, as it is not upregulated by *LBD3* or *LBD11* according to our RNA-seq data. The backbone vector pGreen_dualluc_ORF_sensor^50^ (Addgene No. 55207) was modified in two steps. First, a stop codon and *NOS* terminator (*TAA-NOS*) were inserted directly downstream of the Rluc coding sequence using PstI/AvrII digestion and ligation. Next, the original *NOS* promoter upstream of *Rluc* was removed via AclI/SalI digestion and replaced with a multiple cloning site flanked by the *PLL18* promoter, which was subsequently substituted with *PLL22* or *PLL20* promoters via BamHI/NheI cloning.

For *PLL19* and *PLL26*, which could not be cloned via restriction digestion, reporter constructs were generated using the MultiSite Gateway system. In this system, the *Rluc-NOS* sequence was cloned into the entry vector p221z-BsaI-ccdB-BsaI, and the *CaMV35S*–*Fluc*–*NOS* terminator sequence was cloned in reverse orientation into p2R3z-BsaI-ccdB-BsaI using Golden Gate cloning. The LR reaction was then performed by integrating *1R4a-pPLL19* or *1R4a-pPLL26* in the first box, *221z-Rluc-NOS* in the second box and *2R3z-NOS-Fluc-35S* in the third box into the destination vector pCAM-kan-R4R3 (ref. ^44^).

*Agrobacterium tumefaciens* strain GV3101 (pSoup) was used for *N. benthamiana* leaf infiltration. Overnight cultures grown in LB medium were harvested via centrifugation and resuspended in infiltration buffer containing 10 mM MES (pH 6.0), 10 mM MgCl_2_ and 200 μM acetosyringone, to a final OD_600_ of 0.5. Effector and reporter cultures were mixed in a 1:1 ratio for co-infiltration. For mock controls, reporter cultures were mixed with infiltration buffer lacking the effector construct.

*Agrobacterium* mixtures were incubated at room temperature for one hour prior to infiltration. Each construct combination was infiltrated into four leaves of five-week-old *N. benthamiana* plants. Leaf discs were collected from four infiltrated leaves per sample at three days post-infiltration and immediately frozen in liquid nitrogen. All assays were performed with four to five biological replicates. *Rluc* and *Fluc* activities were measured using the Dual-Luciferase Reporter Assay System (Promega), following the manufacturer’s instructions with a modified reagent volume: 10 μl of sample solution, 25 μl of LAR reagent and 25 μl of Stop & Glo reagent were used for each measurement. Luminescence was detected using a BioTek Synergy H1 microplate reader (BioTek). Data were calculated as the ratio of *Rluc* to *Fluc* activity and normalized to the *Rluc/Fluc* ratio of the mock control.

### Expression and purification of recombinant enzyme in *E. coli*

Signal peptides were predicted using SignalP v.6.0 (ref. ^51^). For protein expression, the predicted signal peptides and potential glycosylation sites^22^ were excluded from the coding sequences (Extended Data Fig. 10a). Initial attempts to express *Arabidopsis* PLL19, PLL22 and PLL26 in *E. coli* using His- or GST-tags failed to yield soluble proteins. Considering the codon bias in *E. coli*, the sequences of *PLL18*, *PLL19*, *PLL22* and *PLL26* were then codon-optimized for *E. coli* expression using the IDT Codon Optimization Tool (https://eu.idtdna.com/pages/tools/codon-optimization-tool), and the optimized sequences are listed in Supplementary Table 9. The predicted molecular weight of the codon-optimized PLL proteins is approximately 41.5 kDa. After purification and western blotting, the recombinant PLL protein appeared as a band near 40 kDa. This difference could be explained by the different migration rate between the molecular weight marker and the PLL proteins on SDS–PAGE gel. Alternatively, the recombinant protein might have undergone partial proteolytic cleavage, similar to what has been reported for a functional pectate lyase from *Zinnia*^22^.

Each codon-optimized gene was synthesized and cloned into the pET28a(+) vector (Novagen) by Twist Bioscience, incorporating an in-frame C-terminal 6×His tag using the NcoI and XhoI restriction sites. The empty pET28a(+) vector served as a negative control. Constructs were transformed into *E. coli* BL21(DE3)pLysS cells and plated on LB agar containing 50 mg ml^−1^ kanamycin.

Transformed colonies were inoculated into LB medium with kanamycin and grown overnight at 37 °C. The overnight culture was diluted to an OD_600_ of 0.1–0.2 in 500 ml of fresh LB medium with kanamycin and incubated at 37 °C until reaching an OD_600_ of ~0.5. Protein expression was induced by adding 0.4 mM IPTG, followed by further incubation at 37 °C until the culture reached an OD_600_ of ~1.0.

Cells were harvested via centrifugation at 4,000 *g* and resuspended in 8 ml of lysis buffer (50 mM Tris-HCl, pH 7.5; 100 mM NaCl; 0.2 mg ml^−1^ lysozyme; EDTA-free protease inhibitor tablet (Pierce); 0.1% *n*-dodecyl α-D-maltoside). Cells were lysed via sonication on ice using a BANDELIN HD 2070. Lysates were clarified via centrifugation at 12,000 *g*, and the resulting supernatant was subjected to His-tag affinity purification using Ni Sepharose 6 Fast Flow beads (Cytiva). For purification, 100 µl of beads were first washed with five volumes of wash buffer (50 mM Tris-HCl, pH 7.5; 500 mM NaCl; 40 mM imidazole). The cleared protein lysate was then incubated with the washed beads for 1 h at room temperature on a rotor. After incubation, the beads were washed five times with wash buffer, and proteins were eluted in 4×50 µl of elution buffer (50 mM Tris-HCl, pH 7.5; 500 mM NaCl; 500 mM imidazole).

Protein concentrations were determined via Bradford assay. Bio-Rad Protein Assay Dye was diluted 1:5 in distilled water. A standard curve was generated using BSA (1 µg µl^−1^) with 0, 1, 2, 5, 10 and 15 µg of protein in 20 µl total volume. For samples, 5 µl of elution fractions was mixed with 15 µl of H_2_O. 980 µl of diluted Bradford reagent was added to all samples including the standard curve, and absorbance at 595 nm was measured with a spectrophotometer.

Approximately 15 µg of purified protein was loaded per lane for SDS–PAGE and western blotting. Gels were either transferred for western blot or stained with Coomassie Brilliant Blue for 1 h, followed by destaining and imaging via scanner.

### Western blot analysis

Following SDS–PAGE, proteins were transferred to a PVDF membrane. The membrane was blocked with 5% BSA in TBST (1× TBS + 0.05% Tween-20) for 1.5 h at room temperature, then incubated overnight at 4 °C with mouse anti-6×His primary antibody (Abcam) diluted 1:3,000 in 1% BSA in TBST. The membrane was washed five times in TBST, then incubated with HRP-conjugated anti-mouse IgG secondary antibody (Bio-Rad, No. 1706516) diluted 1:10,000 in 1% BSA in TBST for 1 h at room temperature. After washing three times in TBST and two times with TBS, the membrane was incubated with ECL Prime reagents (Cytiva), and chemiluminescence signals were visualized with the iBright FL1500 imaging system (Invitrogen). After western blot detection, the same membrane was stained with Amido Black to visualize total protein loading.

### Pectate lyase activity assay

Pectate lyase activity was measured using a pectate lyase assay kit (Arigo, No. ARG82026) according to the manufacturer’s instructions. The concentration of purified protein was determined as described above, and equal amounts of total protein were used for both the pET28a(+) and pET28a(+)-PLL18-co samples. Each reaction consisted of 90 µl of substrate solution mixed with 10 µl of protein sample and incubated at 50 °C for 15 min. After incubation, 100 µl of stop solution was added, and the mixture was centrifuged at 5,000*g* for 5 min. A 100 µl aliquot of the supernatant was transferred to a 96-well UV-transparent microplate, and absorbance was measured at 235 nm using a BioTek Synergy H1 microplate reader. Heat-inactivated (boiled) samples were used as negative controls. Enzyme activity was calculated on the basis of absorbance differences using the formula provided by the manufacturer: pectate lyase (U ml^−1^) = {[(OD_sample_ − OD_control_)/(*ε* × *d*) × *V*_total_]/*V*_sample_}/*T*, where *ε* = 5.2 × 10^3^ l mol^−1^ cm^−1^ (molar extinction coefficient); *d* = 0.3 cm (optical path length of a 96-well plate); *V*_sample_ = 0.01 ml; *V*_total_ = 0.2 ml; *T* = 15 min (reaction time). One unit (U) of pectate lyase activity is defined as the amount of enzyme required to release 1 nmol of unsaturated oligogalacturonic acid per minute under the assay conditions.

### RT-qPCR

Roots from nine-day-old plants were treated for one day with either a mock solution or 1 μM BAP. Main root segments, located 0.5–1.5 cm below the root–hypocotyl junction, were collected with four biological replicates. RNA isolation, cDNA synthesis and the qPCR program followed the protocol described previously^17^. Relative expression levels were calculated using the 2^−ΔΔCt^ method^52^ and normalized to the reference genes *UBQ10*, *ACT2* and *TIP41*.

### AFM

Roots were fixed in 4% PFA in 1× PBS for one hour under vacuum or overnight at 4 °C. After fixation, the samples were rinsed twice with 1× PBS, aligned and sectioned in stiff 4% agar. A Leica vibratome was used to obtain 200-μm cross sections for AFM analysis. To enable comparative analysis, samples intended for side-by-side comparisons were embedded in the same section within separate bundles. For AFM measurements, vibratome sections were mounted on glass microscope slides in 1× PBS containing 0.55 M mannitol.

High-resolution images and mechanical maps of the samples were collected with a NanoWizard IV XP BioScience atomic force microscope (JPK-Bruker) mounted on top of an Olympus IX 73 inverted optical microscope (Olympus Corporation). The samples were scanned using Quantitative Imaging Advanced mode and ScanAsyst-Fluid probes with a nominal tip radius of 20 nm (Bruker). The probes were previously calibrated by determining their deflection sensitivity against a clean glass microscope slide and applying the thermal noise method, resulting in spring constants within the range 0.8−1.0 N m^−1^.

AFM images and data were analysed using the JPK Data Processing software (JPK-Bruker). To obtain Young’s modulus maps from the indentation force curves recorded at every pixel of the images, batch processing of the Quantitative Imaging data files was performed according to the instructions provided in the user manual. The analysis involved baseline correction, vertical tip position adjustment and the sequential application of three reference force-height operations. The indentation force curves were analysed using the Hertz model, assuming a Poisson’s ratio of 0.5. The same parameters were applied to the sample being compared.

### Immunofluorescence

For the WT, *pll19* *pll22* *pll26* and *lbd3* *lbd4* *lbd11*, roots from ten-day-old plants were used. For the *LBD11* inducible overexpression line, roots from ten-day-old plants were collected with either one or two days of induction, as well as with a two-day mock treatment. Roots were fixed overnight at 4 °C in 4% PFA in PBS, followed by two washes in cooled 1× PBS, then embedded in small blocks of 4% agar. To enable comparative analysis, samples intended for side-by-side comparisons were embedded in the same section within separate bundles. 200-μm vibratome sections were taken approximately 5 mm below the hypocotyl–root junction. Sections were blocked with MPBS (1% (w/v) milk and 0.1% (v/v) TWEEN 20 in 1× PBS) for 30 min at room temperature. Primary antibody LM19 (PlantProbes) was applied at a 1:200 dilution in MPBS overnight at 4 °C or for 2 h at room temperature, followed by five washes in MPBS. Alexa Fluor 488-conjugated goat anti-rat IgM (Thermo Fisher Scientific, A-21212) was applied as the secondary antibody at a 1:400 dilution in MPBS, incubated overnight at 4 °C or for 2–4 h at room temperature, followed by five washes in 1× PBS.

Confocal imaging was performed in 1× PBS containing 1 µl ml^−1^ SR2200 using a Leica Stellaris 8 confocal microscope with a ×63 objective lens. Immunofluorescence images represent maximal projections of five to seven consecutive optical sections captured from the start to the end of the signal in the *z*-direction. For quantifying signal intensity in the cell wall region, Fiji software was used. A max-intensity *z*-projection was first applied, and the cell wall was isolated by adjusting the threshold in the SR2200 cell wall staining image. Although the threshold could vary between experiments, it was always kept consistent for each experimental sample and its corresponding control. Immunofluorescence intensity in the cell wall region was then quantified on the basis of the isolated cell wall region from the cell wall staining image.

### Monosaccharide analysis via trimethylsilyl derivatization

For the WT and mutants, roots from 12-day-old WT, *pll19* *pll22* *pll26* and *lbd3* *lbd4* *lbd11* plants were used. The *lbd3* *lbd4* *lbd11* and *pll19* *pll22* *pll26* mutants were processed in separate rounds, each with a corresponding WT sample as a reference. For the *LBD11* inducible overexpression line, roots from 12-day-old plants treated for two days with either 5 μM β-17-estradiol or a mock solution were used.

Each sample included four biological replicates. For each replicate, approximately 7 cm of the root region from the root–hypocotyl junction, excluding visible lateral roots, was collected from around 70 individual plants and placed into a 2 ml Eppendorf tube. Dried roots were ground into powder, which was then incubated with 80% ethanol for 30 min at room temperature, followed by chloroform:methanol (1:1) for 10 min. The remaining material was dried, weighed and incubated overnight at 37 °C in a starch digestion solution (1 ml per 10 mg of material; 0.1 M potassium phosphate buffer, pH 7.0, containing 10 mM NaCl, 1 μl of 0.01% sodium azide and 10 units of α-amylase (Roche, 10102814001). After removal of starch, the samples were washed with water and acetone and dried overnight.

500 μg (±5%) of dry fine powder and 30 μg of inositol, used as an internal standard, together with standards of nine monosaccharides (Ara, Rha, Fuc, Xyl, Man, Gal, Glc, GalA and GlcA (Merck KGaA), each at 10, 20, 50 and 100 μg) were methanolized and derivatized, and its silylated monosaccharides were separated in gas chromatography/mass spectrometry (7890A/5975C; Agilent Technologies) according to Sweeley et al.^53,54^ and Latha Gandla et al.^55^.

Raw data MS files from gas chromatography/mass spectrometry analysis were converted to NetCDF format in Agilent Chemstation Data Analysis (v.E.02.00.493) and exported to the RDA (v.2016.09; Swedish Metabolomics Centre). Data pretreatment procedures, such as baseline correction and chromatogram alignment, peak deconvolution and peak integration followed by peak identification were performed in RDA. 4-*O*-methylglucuronic acid was identified according to Chong et al.^56^. The amount of each sugar was normalized on the basis of inositol and calculated according to the standards. The mole percent (Mol %) is determined by dividing the µmoles of a specific monosaccharide by the total µmoles of all monosaccharides. Analyses were done in the Biopolymer Analytical Platform at Umeå Plant Science Centre (UPSC)/Swedish University of Agricultural Sciences (SLU), Sweden.

### Statistical analysis

R (v.4.3.3) was used for plotting. Statistical analyses were conducted using IBM SPSS Statistics (v.29.0.2.0). Normality of distribution was assessed using Shapiro–Wilk tests; for normally distributed data, a two-tailed Student’s *t*-test was applied, while non-normally distributed data were analysed with a two-tailed Mann-Whitney *U*-test. For ANOVA tests, Levene’s statistic was used to test the homogeneity of variances. Significant differences, indicated by different letters, were determined at an *α* level of 0.05 using one-way ANOVA.

### Reporting summary

Further information on research design is available in the Nature Portfolio Reporting Summary linked to this article.

## Supplementary information

Supplementary Information Supplementary Figs. 1 and 2 and uncropped scans of gels and blots.

Reporting Summary

Supplementary Tables Supplementary Tables 1–9.

Supplementary Data 1 Source data for Supplementary Fig. 2.

## Source data

Source Data Fig. 1 Statistical source data.

Source Data Fig. 2 Statistical source data.

Source Data Fig. 3 Statistical source data.

Source Data Fig. 4 Statistical source data.

Source Data Fig. 5 Statistical source data.

Source Data Extended Data Fig. 6 Statistical source data.

Source Data Extended Data Fig. 7 Statistical source data.

Source Data Extended Data Fig. 8 Statistical source data.

Source Data Extended Data Fig. 10 Uncropped blots and gels.

## Data Availability

All lines involved in this study are available upon reasonable request from the corresponding author. Raw RNA-seq data have been deposited in the Sequence Read Archive under accession number PRJNA1288429. Source data are provided with this paper. All other data supporting the findings of this study are available in this article and its supplementary information.
