# Supplementary material for: Cambium LBDs promote radial growth by regulating PLL-mediated pectin metabolism
Source: Nat Plants. 2025 Nov 14;11(12):2565–80. doi: 10.1038/s41477-025-02151-1 (PMC12711582; doi:10.1038/s41477-025-02151-1)
Supplement: Supplementary file 1 — Supplementary Figs. 1 and 2 and uncropped scans of gels and blots. [file 41477_2025_2151_MOESM1_ESM.pdf]

nature plants

Supplementary information https://doi.org/10.1038/s41477-025-02151-1

Cambium LBDs promote radial growth by regulating PLL-mediated pectin metabolism

In the format provided by the authors and unedited

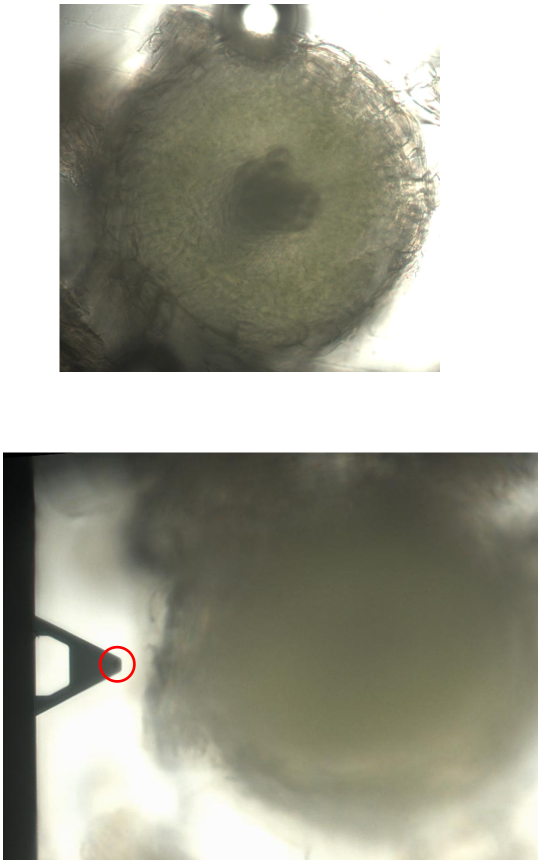

Supplementary Figure 1: The AFM experiment setup.

A representative vibratome section (upper) used for the AFM experiment, and an image (lower) showing a ScanAsyst-Fluid probe used for scanning the section in PBS solution. The red circle indicates the location of the probe tip.

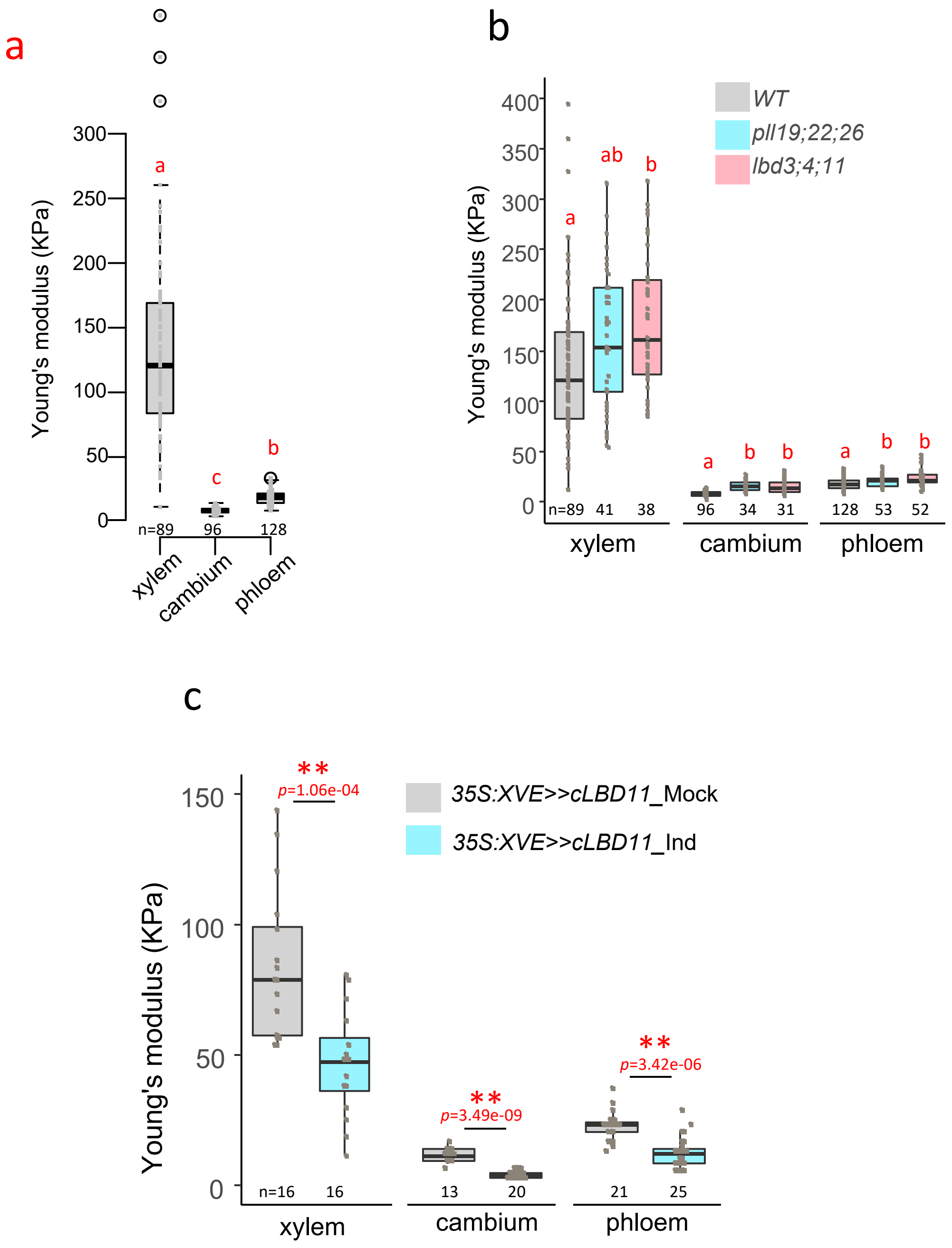

Supplementary Figure 2: Quantification of the AFM data.

a, Quantification of Young's modulus across the xylem, cambium, and phloem in WT roots; the same WT dataset is also shown in panel b for comparison. b, c, Quantification of Young's modulus across the same tissues from the experiments shown in Fig. 5h and 5j. A separate ANOVA test was performed for phloem, cambium and xylem regions. Significant differences, indicated by different letters, were determined at an alpha level of 0.05 using a one-way ANOVA with Tukey's post hoc test (for equal homogeneous variance) or Tamhane's post-test (for unequal variance). Exact p-values for each comparison are provided in Source Data. A two-tailed Mann-Whitney test was used in panel (c). $*p < 0.05$; $**p < 0.01$. n and grey dots in the boxplot represent individual measurements from the cell wall region of the corresponding cell type. Boxes represent the first (Q1) and third (Q3) quartiles, with the line inside indicating the median. Data are presented as boxplots showing the median (center line), the interquartile range (IQR, box boundaries: 25th and 75th percentiles), and whiskers extending to the minimum and maximum values within 1.5 × IQR of the quartiles.
