# Supplementary figures and images for: Cambium LBDs promote radial growth by regulating PLL-mediated pectin metabolism
Source: Nat Plants. 2025 Nov 14;11(12):2565–80. doi: 10.1038/s41477-025-02151-1 (PMC12711582; doi:10.1038/s41477-025-02151-1)

Source\_Data\_Extended\_Data\_Fig\_10

Uncropped scans of gels and blots

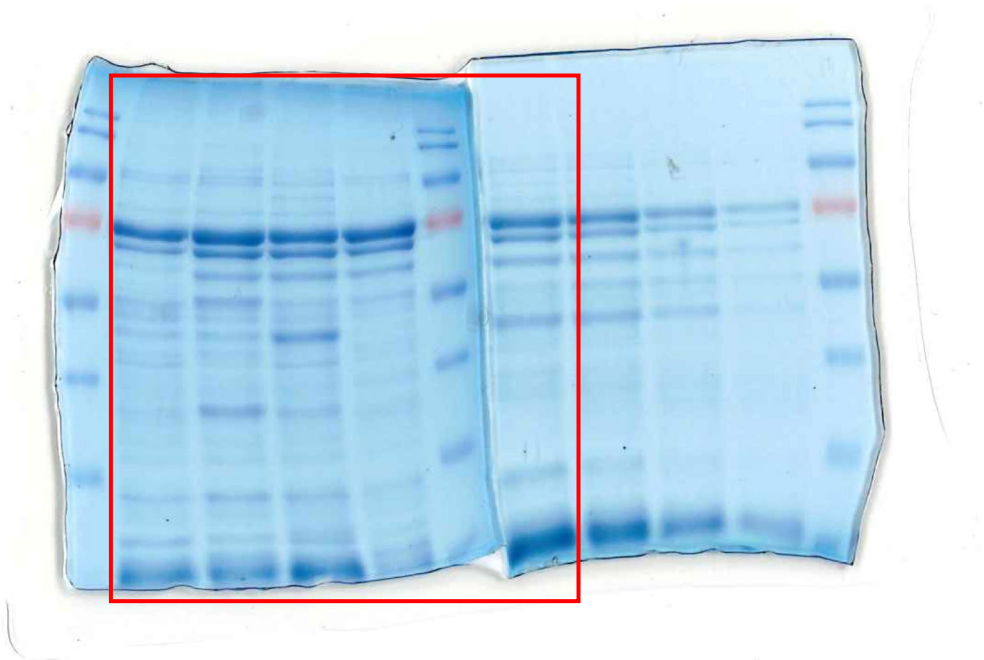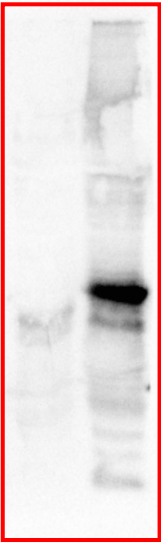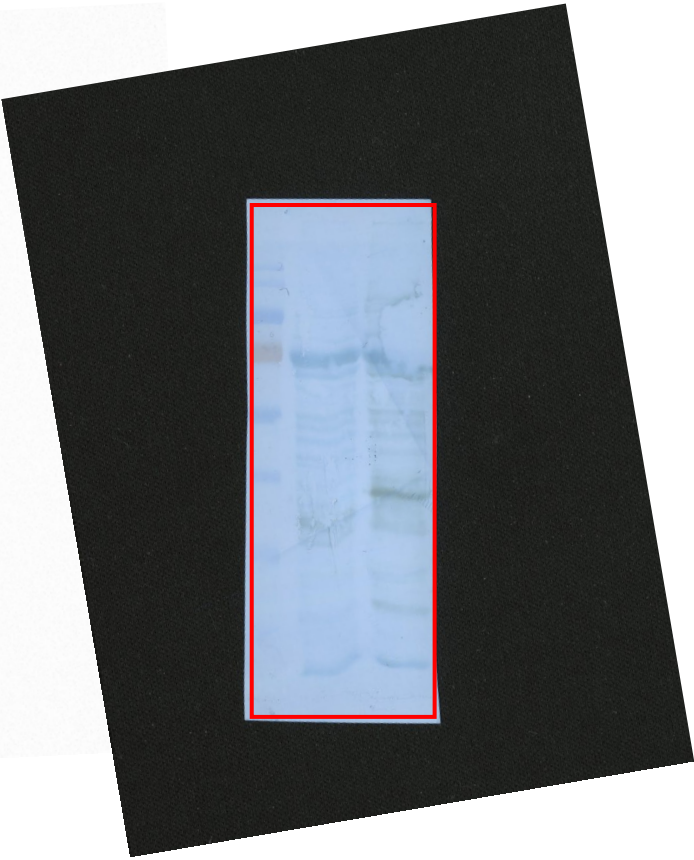

Supplement: Supplementary file 13 — Uncropped blots and gels. [file 41477_2025_2151_MOESM13_ESM.pdf]
